# The Long Goodbye: Finally Moving on from the Relative Potency Approach to a Mixtures Approach for Polycyclic Aromatic Hydrocarbons (PAHs)

**DOI:** 10.3390/ijerph19159490

**Published:** 2022-08-02

**Authors:** Lynne T. Haber, Alison M. Pecquet, Melissa J. Vincent, Louise M. White

**Affiliations:** 1 Department of Environmental and Public Health Sciences, College of Medicine, University of Cincinnati, 160 Panzeca Way, Cincinnati, OH 45267, USA; alison.pecquet@syngenta.com (A.M.P.); melissa.vincent@cardno.com (M.J.V.); 2 Syngenta AG, Greensboro, NC 27409, USA; 3 ChemRisk, Cincinnati, OH 45242, USA; 4 Environmental Health Program, Health Canada, Halifax, NS B3J 3Y6, Canada; louisewhite@eastlink.ca

**Keywords:** PAHs, PEF, RPF, potency, mixtures

## Abstract

For the past several decades, a relative potency approach has been used to estimate the human health risks from exposure to polycyclic aromatic hydrocarbon (PAH) mixtures. Risk estimates are derived using potency equivalence factors (PEFs; also called relative potency factors [RPFs]), based on the ratio of selected PAHs to benzo[a]pyrene (BaP), expressed qualitatively by orders of magnitude. To quantify PEFs for 18 selected carcinogenic PAHs, a systematic approach with a priori and dose response criteria was developed, building on draft work by the US EPA in 2010 and its review by US EPA Science Advisory Board (SAB) in 2011. An exhaustive search for carcinogenicity studies that included both target PAHs and BaP with environmentally relevant exposure routes found only 48 animal bioassay datasets (mostly pre-1992 based on skin painting). Only eight datasets provided adequate low-response data, and of these only four datasets were appropriate for modeling to estimate PEFs; only benzo[b]fluoranthene and cyclopenta[c,d]pyrene had a PEF that could be quantified. Thus, current knowledge of PAH carcinogenicity is insufficient to support quantitative PEFs for PAH mixtures. This highlights the long-acknowledged need for an interdisciplinary approach to estimate risks from PAH mixtures. Use of alternative and short-term toxicity testing methods, improved mixture characterization, understanding the fate and bioavailability of PAH mixtures, and understanding exposure route-related differences in carcinogenicity are discussed as ways to improve the understanding of the risks of PAHs.

## 1. Introduction

Polycyclic aromatic hydrocarbons (PAHs) are a ubiquitous group of complex hydrocarbons and are commonly found in the environment as mixtures (rather than single PAHs). Petrogenic PAHs are naturally present in coal and crude oil, and remain within refined coal, coal tar, coal-tar pitch, and petroleum products. Pyrogenic PAHs are formed as the result of incomplete combustion, and are therefore found in soot/smoke, diesel particulate matter, and some foods cooked at high temperature.

The International Agency for Research on Cancer (IARC) has found unsubstituted PAHs and certain PAH mixtures to be either carcinogenic (Group 1), probably carcinogenic (Group 2A) or possibly carcinogenic (Group 2B) to humans, based on human and/or experimental animal data. For example, occupational oral, dermal, and inhalation exposure to PAH mixtures formed in industrial processes, such as coal gasification, coke production, coal tar distillation, and paving and roofing with coal tar pitch, are considered carcinogenic to humans [1].

However, typical laboratory analyses do not identify all individual PAHs within a mixture, and it is not feasible to determine the toxicity or carcinogenicity of every one of the large numbers of PAH mixtures to which people may be exposed. As a more manageable approach, potency equivalence factors (PEFs), also known as relative potency factors (RPFs), have been determined for a small group of PAHs commonly found in PAH mixtures to which humans are exposed.

Carcinogenic PEFs are calculated by expressing the carcinogenic potency of the target PAH relative to that of a more well-studied PAH, typically benzo[a]pyrene (BaP). The carcinogenic potency of PAH mixtures is characterized by multiplying the concentrations of select PAHs in environmental media by the relevant PEFs and summing the resulting values. Many of the currently available PEFs were derived based on the professional judgement of an expert panel following semi-quantitative evaluation of the carcinogenicity data (e.g. [2], as cited in [3]). Most published PEFs are therefore not exact representations of cancer potency, and are instead expressed as orders of magnitude (i.e., are restricted to factors of 10 or 0.1), reflecting the uncertainty in evaluating relative potency of PAHs.

Almost two decades ago, the United States Environmental Protection Agency (US EPA) [4] PAH workshop concluded that the relative potency approach was the least scientifically defensible approach to estimate the toxicity of PAH mixtures, and that any approach that utilizes the toxicity of a mixture as a whole (rather than the toxicity of selected components) is preferable. Such whole-mixture approaches have also long been deliberated ([3,5], as cited in [6,7,8,9]). Due to this, the US EPA’s Science Advisory Board (SAB; [10]) recommended that the US EPA develop a mixtures approach in the future. In a whole mixtures approach, the potency of the mixture of concern is estimated by applying the potency for a surrogate mixture or sufficiently similar mixture. The US EPA [4] noted that a whole-mixture approach would be preferred when appropriate data are available, as this approach would not require substantial information on the individual components of the mixtures, their toxicological potencies or their modes of action, nor does it require any assumptions with respect to component interactions [4]. However, these whole-mixture approaches for PAHs are limited by the diversity of PAH mixtures in the environment; relatively few PAH-containing mixtures have been characterized with respect to cancer potency. Consequently, many regulatory agencies currently employ a PEF-based approach to assess the carcinogenicity of PAH mixtures (see Table 1), including Health Canada [11], the US EPA [12,13], California EPA [14], Netherlands National Institute for Public Health and the Environment [15], and United Kingdom Environment Agency [16]. In 2010, the Canadian Council of Ministers of the Environment (CCME) evaluated the relative potency approach as a tool to derive soil quality guidelines (SQGs) for carcinogenic PAHs, and concluded that adoption of PEFs was necessary because there was no other viable approach [11].

In 2010, the US EPA’s Integrated Risk Information System (IRIS) Program released a draft report evaluating the relative potency approach for PAH mixtures [17]. The EPA evaluation was based on a comprehensive literature search for studies that reported quantitative dose–response data for both individual PAHs and BaP in rodent carcinogenicity or cancer-related assays (i.e., genotoxicity, DNA repair, and cell transformation); the studies evaluated employed exposure by either environmentally relevant (oral, dermal) or parenteral (intraperitoneal, subcutaneous, and lung implantation) routes. The final analyses included tumor data for 74 carcinogenic and non-carcinogenic unsubstituted PAHs and the resulting PEFs were developed based on both carcinogenicity and genotoxicity data.

In their review of the US EPA draft report, the US EPA SAB [10] agreed with the PEF concept in principle, but recommended that the US EPA focus only on carcinogenicity studies that used environmentally relevant exposure routes (i.e., oral, dermal, or inhalation). The SAB Panel also strongly supported the use of cancer bioassay data when determining the PEF for a given PAH; although data addressing cancer-related endpoints were considered useful for supporting the results of carcinogenicity studies, the SAB Panel did not recommend relying only on cancer-related endpoint data when determining PEFs. The SAB also made several specific recommendations regarding the methods for the quantitative analyses.

The purpose of this analysis was to build on the premise of the draft 2010 US EPA report, while addressing only a targeted subset of 18 suspected high-potency carcinogenic PAHs and one alkylated carcinogenic PAH. The PAHs evaluated were those that are typically found in soil or sediment at contaminated sites and for which Canada [11] had previously adopted PEFs; we also evaluated selected PAHs for which there is evidence that their potency is equal to or greater than that of BaP. The US EPA [17] literature review was also updated and the SAB recommendations [10] regarding study selection and dose–response analyses were adopted.

## 2. Materials and Methods

**Figure 1:**
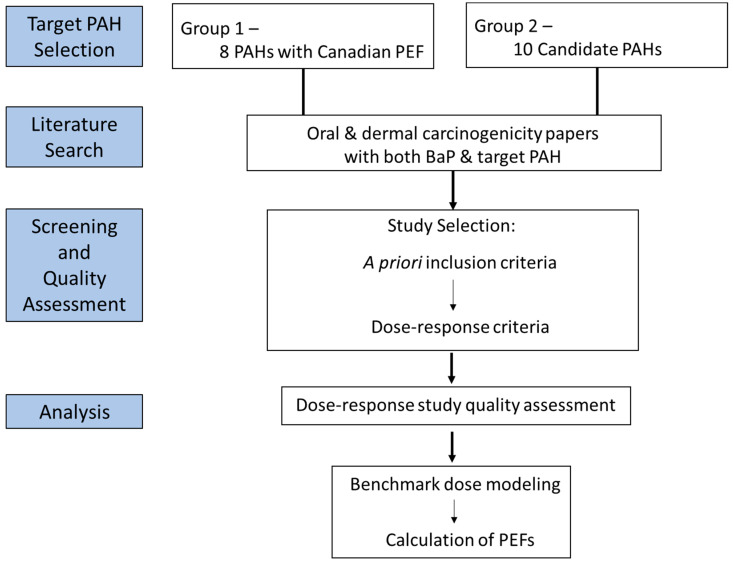
Conceptual Model of Approach to Data Gathering and Analysis.

A conceptual model illustrating our process for selection of candidate PAHs, literature searching, screening and quality assessment of studies, dose–response modeling, and PEF derivation is provided in Figure 1.

### 2.1. Selection of Candidate PAHs

The initial analysis focused on eight target carcinogenic PAHs for which Canadian PEF values are currently available, and on the comparator chemical BaP (Table 1). Subsequently, 13 additional candidate carcinogenic PAHs, including alkylated PAHs, were also considered (Table 2); the criteria for choosing these 13 candidates are listed in Table 3. The ultimate goal was to identify less commonly analyzed PAHs for which the published PEF values are similar to or exceed that for BaP (i.e., are greater than 1). Selection of these 13 PAHs for analysis considered the availability of a published IARC classification of carcinogenicity for the PAH [1], PEFs published by the US EPA [17], and/or other PEFs from the literature. A target PAH was included if the IARC concluded the PAH was possibly carcinogenic (e.g., benz[c]phenanthrene) even if the PAH had no published PEFs. A candidate PAH was also included if the IARC concluded there was inadequate evidence for carcinogenicity in humans, but PEFs had nonetheless been published (e.g., dibenz[a,c]anthracene, dibenzo[a,e]fluoranthene, and dibenzo[a,e]pyrene). Of the few alkylated PAHs reviewed by the IARC [1], only 5-methylchrysene has been classified as possibly carcinogenic; for all other PAHs considered, the evidence was deemed inadequate to evaluate carcinogenicity in humans. Another critical requirement was the availability of laboratory standards for chemical analysis of candidate PAHs in tissues and environmental media, since the goal was to develop PEFs for practical use at waste sites.

**Table 1:** Survey of Potency Factors for the Most Commonly Examined Polycyclic Aromatic Hydrocarbon (PAH).

Polycyclic Aromatic Hydrocarbon (PAH)	Short Form	CAS Number	PEF Relative to BaP ^2^					
			IARC (2010) Class ^1^	WHO/IPCS (1998) ^2^	CCME PEF ^3^ (2010)	EPA RPF ^4^ (1993)	IRIS US EPA RPF (2010) ^5^	Range in Published PEFs (Long et al., 2017) ^6^
Benzo[a]pyrene	BaP	50-32-8	1	1	1	1	1	1
Benz[a]anthracene	BaA	56-55-3	2B	0.1	0.1	0.1	0.2	0.0005–0.145
Benzo[b]fluoranthene	BbF	205-99-2	2B	0.1	0.1 ^7^	0.1	0.8	0.06–0.62
Benzo[j]fluoranthene	BjF	205-82-3	2B	0.1		-	0.3	-
Benzo[k]fluoranthene	BkF	207-08-9	2B	0.1		0.01	0.03	0.01–0.1
Benzo[g,h,i]perylene	BghiP	191-24-2	3	0.01	0.01	-	0.009	0.01–0.022
Chrysene	CH	218-01-9	2B	0.1	0.01	0.001	0.1	0.001–0.10
Dibenz[a,h]anthracene	DBahA	53-70-3	2A	1	1	1	10	0.69–5
Indeno[1,2,3-cd]pyrene	IP	193-39-5	2B	0.1	0.1	0.1	0.07	0.017–0.278

^1^ IARC Classification (2010) [1]: 1 = carcinogenic to humans (applies to BaP); 2A = probably carcinogenic to humans; 2B = possibly carcinogenic to humans; 3 = not classifiable as to its carcinogenicity to humans. ^2^ Kalberlah et al. (1995) [2] in WHO/IPCS [3] was the originating source for CCME (2010) [11] PEFs with modifications; see footnote 5. ^3^ Potency Equivalence Factors (PEFs) used by Canada and others (e.g., CCME, 2010) [11]. ^4^ Relative Potency Factors (RPFs) used by US EPA (e.g., 1993 [12] and 2010 [17]). RPFs and PEFs are synonymous. ^5^ Draft IRIS (US EPA 2010) [17] asks to neither cite nor quote. Potency only provided for comparative purposes. ^6^ Long et al., 2017 [18]. Table S2: A literature review was conducted to identify the lowest and highest published PEFs for each compound. See Supplemental Tables for original source references. ^7^ BbF and BjF closely co-elute in past laboratory analyses and therefore were reported as a combined sum of isomers. CCME elected to sum all three potentially co-eluting benzofluoranthenes (i.e., benzo[b+j+k]fluoranthene) for their PEFs. Although US EPA does not include B[j]F in their priority pollutant list, in practical terms both B[b]F and B[j]F are analytically indistinguishable. Current laboratory analysis can now easily separate BbF and BjF (Ruffolo et al., 2017, pers. comm.) [19].

Potential candidates for PEF evaluation included the 10 PAHs listed in Table 2 and benzo[j]aceanthrylene, benzo[l]aceanthrylene, and 4H-cyclopenta[def]chrysene (as identified by [19]). The final check for the candidate list consisted of a literature search to ascertain whether candidate PAHs were found in the environment in media such as soil and therefore relevant to oral exposure. Benz[j]aceanthrylene, benz[l]aceanthrylene, and 4H-cyclopenta[def]chrysene were excluded from the analyses because they are generally detected in particulate air pollution in urban environments (e.g., diesel exhaust, cigarette smoke) and there was little, if any, published evidence that they have been detected in soil or sediments. Our final list included 18 target PAHs plus BaP as the comparator.

Abbreviations for the target PAHs used throughout this paper can be found in Table 1 and Table 2.

### 2.2. Literature Searches

The primary search criterion for our study was that the experimental design include concurrent testing of the target PAH and the comparator PAH using a physiological route of exposure, as recommended in the draft US EPA [17] document. Concurrent testing of the target and comparator PAH minimized the impact of inter-laboratory variability on the estimated relative potency. The SAB [10] supported the US EPA stipulation that BaP be tested concurrently with the target PAH, but also allowed for the possibility of using another PAH as a comparator if a high-quality PEF had been or could be derived. Although we identified other potential comparators, none had adequate dose–response data, and we therefore restricted screened studies to those with BaP as the comparator.

For the initial analyses, we utilized the results of the US EPA [17] literature search to identify publications that concurrently addressed the carcinogenicity of the 18 target PAHs and BaP in animal bioassays. Additional searches of the PubMed and TOXNET databases were conducted using broad search criteria to identify carcinogenicity studies published from January 2009 through January 2016 for the PAHs listed in Table 1 (target PAHs), and from January 2009 through February 2018 for the additional PAHs analyzed (Table 2). The US EPA [17] did not provide a PEF for benzo[c]phenanthrene, nor for any alkyl-PAHs such as 5-methylchrysene, and a targeted search was undertaken for studies addressing these two PAHs. Authoritative review documents from the WHO/IPCS [3], IARC [1], NTP [23], US EPA [17], and CalEPA OEHHA [24,25] were also examined to identify additional data relevant to the 18 target PAHs. Additional tree searching (e.g., searching for additional articles by authors of key publications and review of references cited in the authoritative reviews) was also performed to identify any additional studies. Publications that included only cancer-related endpoints (e.g., in vitro or in vivo genotoxicity data) were not selected for further analyses, although several studies investigating DNA adducts were retrieved to confirm that the study did not include new tumor data.

### 2.3. Study Selection

Based on the SAB [10] recommendations, we developed both a priori criteria for study inclusion and dose–response criteria for the ability to derive a PEF (Figure 1; Table 3) to select tumor studies for dose–response modeling and relative potency calculations.

Within a particular scientific paper, each dose–response experiment was counted as an individual study, such that some papers contained more than one target PAH/BaP study (see Table 4). Complete carcinogenicity and initiation/promotion studies were considered separate studies (Table 4).

#### 2.3.1. A Priori Criteria

The SAB [10] recommended that quality criteria for inclusion of studies for the relative potency calculation be defined and applied a priori to identify studies of sufficient quality.

The first of these a priori criteria addressed the quantification of tumors. The SAB noted carcinogenicity data could include assessment of the incidence of either total or malignant tumors (number of animals with tumors in a population) and/or tumor multiplicity (number of tumors per mouse or per mouse in a specific organ). In the absence of adequate dose–response data for tumor multiplicity, the SAB recommended that only tumor incidence data be used to calculate final PEFs. The SAB also noted that tumor multiplicity and incidence data should not be averaged together. Based on these recommendations, we included only studies with tumor incidence data for both BaP and a target PAH.

Our second a priori selection criterion based on the SAB [10] review comments (Table 3) was that the study used a physiological exposure route (oral, inhalation, or dermal). The SAB [10] noted that the exposure route can impact PAH toxicokinetics and the apparent relative potency of the test compound as compared to BaP.

#### 2.3.2. Dose–Response Criteria

Studies that met the a priori inclusion criteria were further evaluated to determine whether they also met the additional “dose–response criteria” to allow calculation of PEFs (Table 3).

The SAB [10] specifically recommended that studies assessing only a single dose of BaP and/or of the target PAH should be included in PEF analyses only if the BaP tumor incidence was in the low range (50% response or less) and adequate dose–response data were available for the target PAH. The SAB [10] further recommended PEFs be calculated from studies that included multiple doses of BaP and multiple doses of the target PAH. Although the SAB did not explicitly indicate why a less than 50% response was chosen, we considered that a 50% response in single-dose studies or at the lowest non-zero dose of multi-dose studies was a reasonable, if semi-arbitrary, cut-off to ensure that a study captured data in the low-dose region. Applying the criterion to multi-dose studies recognizes the increased uncertainty associated with extrapolation from high doses to the 10% response that was used to characterize carcinogenic potency.

The SAB expressed a preference for calculating overall PEFs only if PEFs were available from more than one experimental dataset. The SAB [10] also suggested that a quality assessment should be completed for each individual study used to derive PEFs, and should include information such as sample size, dosing regime, mortality prior to tumor development, test compound purity, and whether data were derived from tumor incidence or multiplicity data. Where available, we also extracted data regarding the frequency of administration and exposure duration, the control solvent/vehicle, and, when such data were provided, the location of tumors, types of tumors (papillomas, adenomas, carcinomas, etc.), and stage of tumors (benign, malignant). These data related to study design and study quality are presented in Supplemental Table S1, with two exceptions. First, data addressing mortality prior to tumor development were rarely provided, and so are not listed, but were noted for those studies for which they were available. Second, tumor multiplicity data were extracted but not used in the quantitative analyses, and are not included in Table S1.

### 2.4. Benchmark Dose Modeling

Consistent with the SAB guidance [10], the PEF calculation was based on comparison of potencies of the PAH of interest and the comparator PAH (BaP). Potency was calculated using the US EPA’s benchmark dose (BMD) modeling software (BMDS version 3.2, US EPA; available at https://www.epa.gov/bmds accessed on 25 July 2022). Bayesian model averaging (BMA) was applied to all “unrestricted” dichotomous models. The use of multiple models allowed us to evaluate whether the inclusion of additional models would allow fitting of all datasets, including datasets that were not successfully fitted in the draft IRIS report [17]. The BMA approach was chosen to best reflect the uncertainty in the modeling, and allowed us to avoid issues associated with choosing the best model(s). Although written agency guidance for BMA has not yet been developed, experts worldwide have recently reached consensus on this approach: the “power” parameter (β in the BMDS documentation) should not be constrained, but model averaging, using soft constraints with a Bayesian approach, should be used to avoid biologically unreasonable results [49].

As noted by the US EPA [50], where plateauing of the reported dose–response relationship occurred prior to the highest dose(s), high-dose data can be sequentially eliminated from a model as needed in order to improve model fit. This approach was considered biologically reasonable, since responses at the high dose may reflect saturation or different biological processes than those occurring at lower doses. As PEFs are calculated based on the ratio of doses expressed on a mass basis (mg/kg) rather than on a molar basis (mmol/kg), doses were converted to a mass basis if necessary (see Supplemental Tables).

### 2.5. PEF Calculations

The PEF is defined as the ratio of the potency of the target PAH to the potency of the comparator PAH (BaP). The potency can be expressed as the slope of the dose–response curve (where the slope is defined as the benchmark response [BMR] divided by the dose estimated to correspond to a 10% response [BMD_10_]). A BMR of 10% extra risk (or 0.1) is standard when modeling dichotomous data [50,51]. The BMD_10_ was used to calculate the PEF, rather than the lower confidence limit of the BMD_10_ (BMDL_10_), since determining the ratio of BMDLs may be problematic if the studies have different sample sizes [10].

Thus, the PEF was calculated as:

PEF = (0.1/BMD_10_ for target PAH)/(0.1/BMD_10_ for BaP)

= (BMD_10_ for BaP)/(BMD_10_ for target PAH)

## 3. Results

### 3.1. PAH Selection

The 18 target PAHs for our study were included within Group 1 or Group 2 in Table 1 and Table 2. The two groups reflect different times for selection, different sources for selection, and different criteria for selection, as well as different dates for the literature searching (Table 3). Group 1 PAHs are the most frequently measured PAHs in environmental media or tissues (Table 1), while the Group 2 PAHs are less frequently measured and studied (Table 2). All selected target PAHs were unsubstituted, with the exception of a single alkylated PAH, 5-methylchrysene. Once the target PAHs were identified in the selection phase, no further distinction was made between Groups 1 and 2.

The values of the published PEFs for the most commonly measured PAHs presented in Table 1 ranged over almost three orders of magnitude. In general, target PAHs in Table 2 were selected to represent those PAHs for which the published PEFs indicated their potency was equal or greater than that of BaP. Since the target PAHs listed in Table 2 are less frequently measured in environmental media or tissues, it is not surprising that fewer published PEFs were available. Even though benzo[c]phenanthrene (BcPh) appears to have no published PEFs, the IARC [1] considers it possibly carcinogenic to humans. Note that, unlike the work in this paper, the database for developing the published PEFs in Table 1 and Table 2 was not limited to environmentally relevant routes, and included data from intraperitoneal, subcutaneous, and lung implantation studies.

### 3.2. Study Screening

#### 3.2.1. A Priori Criteria

For the target PAHs in Group 1, a total of 830 publications were identified, while 4675 were identified for the Group 2 target PAHs. These publications were then screened to identify in vivo carcinogenicity studies, and the identified papers were retrieved and screened against the a priori criteria (Table 3). The publications that met those criteria are listed in alphabetical order of the first author in Table 4. Common reasons for study exclusion were that the reference did not include a bioassay, the study was not conducted via environmentally relevant route, or did not include BaP or another well-studied PAH as a comparison chemical.

Benzo[a]pyrene was the only appropriate comparator identified. Some Group 2 PAH publications that lacked data for BaP included data for other common PAHs, such as benz[a]anthracene [52], benzo[e]pyrene [53], 4-fluorobenzo[l]fluoranthene [36], or dibenzo[a,l]pyrene [54]. However, no study definitively linked these other potential comparators quantitatively to BaP carcinogenicity and met our a priori criteria.

Twenty-three publications were found that met the a priori criteria. The 23 publications comprised 48 distinct experiments (Table 4). The publication dates ranged from 1935 to 2015, with the majority having been published from 1966 to 1993. Most publications (19 of 23) that met the a priori criteria for selection had already been identified in the US EPA [17] review (Table 4). Three papers were published after the cut-off date for the US EPA’s 2009 literature search. It was not clear why the LaVoie et al. [36] dibenzo[a,l]pyrene study was not identified by the US EPA [17]. Some studies, such as Barry et al. ([26]; BcPh) and Higginbotham et al. ([34]; DBalP), did not meet the US EPA’s stated selection criteria. Others were not selected because the authors did not consider the dose–response relationship for the target PAH to be positive (e.g. [44,48]). In other cases, where papers included both complete carcinogenicity and initiation/promotion studies, it was not clear why the US EPA [17] chose some experimental data within a particular paper while other data were not mentioned (e.g. [33]). Other differences between the PAH/BaP studies selected for our analyses and those selected by the US EPA reflect differences in the selection criteria [17].

Note that US EPA [17] cited [55], who reported that in studies conducted prior to 1966, the compound reported as dibenzo[a,l]pyrene (DBalP) was actually dibenzo[a,e]fluoranthene (DBaeF). Consequently, US EPA [17] listed DBaeF instead of DBalP as was originally assigned by Hoffman and Wynder [35]. Cavalieri et al. [36] also noted the issue regarding incorrect analytical data on DBalP obtained prior to 1968. Before 1966, the chemical listed in the literature as DBalP (1,2,3,4-dibenzopyrene) was in fact dibenzo[a,e]fluoranthene [56]. Cavalieri et al. [36] noted that synthesis of DBalP by Vingiello et al. [57] and Carruthers [58] occurred in 1966; it was subsequently tested in 1968 by subcutaneous injection in mice, and found to induce sarcomas [55]. Devanesan et al. [59] indicated that any carcinogenicity tests prior to 1968 had incorrectly used the weakly active DBaeF instead of DBalP. Based on this information, and similarly to US EPA [17], we list Hoffman and Wynder (1966) as having tested DBaeF rather than DBalP.

We identified at least one study assessing the carcinogenicity of each of the 18 target PAHs that met the a priori criteria. A relatively large number of studies (six to eight) that met the a priori criteria (Table 5 and Table 6) addressed chrysene (CH), cyclopenta[c,d]pyrene (CPP), and dibenzo[a,l]pyrene (DBalP); in contrast, for most PAHs, there were only one to three studies that met the a priori criteria.

#### 3.2.2. Quality of Studies Meeting the a Priori Criteria

In terms of data quality, there was little consistency regarding protocols and dosing approaches across the publications, with a few exceptions. All studies were conducted using female mice from a variety of strains. Except for one experiment using oral exposure (BcFE; [47]), all were dermal exposures (using skin painting), and skin tumors were evaluated. Fifteen of the 23 publications reported the results of a single experiment meeting the a priori criteria, while eight publications reported multiple (2–12) experiments (Table 4). Of the 48 experimental datasets meeting the a priori criteria, 60% were obtained from initiation/promotion experiments, with the remaining 40% from complete carcinogenicity experiments (Table 4).

Acetone was the most common solvent/vehicle for the PAHs. One study used benzene [26], which may have confounded the carcinogenicity assessment; another 12 studies used dioxane [35], while three of the most recent studies used toluene (Supplemental Table S1). The purity of test compounds, whether for the target PAHs, BaP, or the vehicle control, was not specified in several publications (Supplementary Table S1). All of the initiation/promotion experiments used 12-O-tetradecanoylphorbol-13-acetate (TPA) as a tumor promotor, except for Hoffman and Wynder [35], who used croton oil (see Supplemental Table S1).

We noted that only four of 49 dermal experiments stated that mice were cohoused ([32,36,41,42]) and the number of cohoused mice varied from 4 to 10 mice per cage.

In initiation/promotion studies, the reported age of female mice at initiation ranged from four to nine weeks, but was not specified in three studies (Supplemental Table S1). Initiation dosing protocols varied from single doses of the target PAHs to subdoses applied every two days for 20 days. The promotion and total exposure periods also varied; and TPA was applied dermally two or three times per week. Tumor incidence was most frequently based only on papillomas, while four studies did not specify the type of tumor (Supplemental Table S1). An initiation study using DBalP and BaP with no promotion was also included [30].

In complete carcinogenicity studies, the age of the mice at study initiation also varied, as did the dose frequency and exposure period (Supplemental Table S1). There were also inconsistencies and/or uncertainty as to how mice that died during the experiments were handled in the analyses, and whether they were included in estimations of tumor incidence. Most studies sacrificed mice after a set number of weeks of PAH administration; however, in many studies, some of the mice died before the end of an experiment. In several carcinogenicity studies, mice were sacrificed when tumor size was greater than 2 cm, while in others, mice were sacrificed when moribund or dead; other studies did not specify the conditions for pre-termination sacrifice (Supplemental Table S1). In many cases, it was unclear whether mice that died during an experiment had tumors and if so, whether they were included when counting tumor incidence. Siddens et al. [41] was the only publication that identified the number of mice that died tumor-free before the end of 25 weeks, and those that were tumor-free at the end of promotion period were censored when reporting tumor incidence.

Habs et al. [32] terminated their experiment at natural death, increasing the potential for age-related tumors, although this may be of less concern for skin tumors in light of the zero background response in the publication. Generally, papillomas and carcinomas were not reported separately, but summed for determinations of tumor incidence (Supplemental Table S1).

The publications selected based on the a priori criteria had some notable limitations: none discussed the issue of dependence of survival to the end of the experiment on the dose administered; tumor latency was not reported consistently; and the only study that measured tumor incidences for each tumor type and organ tissue was Tilton et al. [45].

#### 3.2.3. Dose–Response Criteria

Only 8 (17%) of the 48 experimental datasets that met the a priori criteria also met the dose–response criteria (Table 6), and all employed dermal exposure. The experiments that did not meet our dose–response criteria included one that did not provide data on incidences of animals with tumors (BcPh), experiments where no response was evident for either the target chemical or BaP (CPP and DBalP), and others where significant mortality occurred before the conclusion of the experiment (CH and 5-MeC) (see Table 5).

More than half (54%) of the datasets that failed to meet the dose–response criteria included only a single dose of both the target PAH and BaP (Table 5). Another eight datasets included a single dose of BaP with two or three doses of the target PAH, while 14% of datasets included two doses of both the target PAH and BaP. A minority of the experiments failing to meet the dose–response criteria had three or more doses of both the target PAH and BaP.

Most of the experiments failing to meet our dose–response criteria did so due to a very high tumor response (>50%) at the single or lowest (in multi-dose studies) tested dose of BaP (Table 5). Of 24 single dose experiments, the response for BaP varied from 65–100%, with the majority exceeding 80%. Only six experiments meeting the dose–response criteria for BaP failed to meet the dose–response criteria for the target PAH (CH, 5-MeC in [33]; CPP in [28]; DBalP in [36,45]). The response at the only or lowest dose tested for target PAHs in studies that failed the dose–response criteria varied from a low of 57% to 100%.

Only eight experimental datasets from four publications assessing five target PAHs (BbF, BjF, BkF, IP, and CPP) met our dose–response criteria (Table 6): five of the eight datasets were from Habs et al. [32]. Two of the eight experiments used initiation/promotion (I/P) designs [28,38] while the remaining six [29,32] were complete carcinogenicity studies. Except for Cavalieri et al. [28], all experiments included at least three doses of both the target PAH and BaP. For four of five PAHs, only a single experimental dataset met the dose–response criteria, while only for CPP were multiple datasets found to be acceptable (Table 6). Four datasets had dose–response data suitable for PEF calculation, three involving CPP and one BbF. Four datasets (BjF, BkF, IP, and [28] study of CPP) were not selected for modeling because of either minimal tumor response or non-monotonic or no dose–response relationships (Table 6).

The four datasets selected for dose–response modeling shared many of the limitations described above regarding data quality. In particular, purity information for the test agent was lacking for some studies, and there was often no indication that measures were taken to avoid ingestion of dermally applied test material by grooming behavior of the mice. In addition, there was little consistency in many of the experimental parameters (Supplemental Table S1).

### 3.3. Dose–Response Modeling and PEF Calculation

Table 7 presents the BMDs and resulting PEFs calculated for the data sets identified as appropriate for modeling. Due to plateauing of the dose–response relationship, the highest BaP dose in Habs et al. [32] was removed in order to obtain adequate fitting. Only one publication [32] had data appropriate for modeling of both BbF and BaP: the resulting PEF was 0.24, rounded to 0.2. Three publications [29,32,38] provided data appropriate for modeling of both CPP and BaP. The resulting PEFs ranged from 0.03 to 0.12 (mean 0.07). For both chemicals, the calculated PEFs were generally consistent with the largely “order of magnitude” PEFs shown in Table 1 and Table 2.

## 4. Discussion

### 4.1. Assessment of Data Supporting the Relative Potency Approach

This study used a systematic approach to develop quantitative PEFs for a select number of carcinogenic PAHs, building upon preliminary work by the US EPA [17] while also incorporating many of the recommendations from the SAB review [10]. It was determined that data supporting the estimation of quantitative PEFs were extremely limited, with most datasets appropriate for assessment of individual PAH potency published between 1966 and 1992. An extensive literature search returned many of the same publications used in previous semi-quantitative evaluations of PEF values (e.g. [3,17]). After a priori screening based on environmentally relevant route of exposure and assessment of carcinogenicity (excluding cancer-related endpoints), fewer studies than used in US EPA [17] were included in our final analyses.

All selected experiments used female mice, and all except one were based on dermal application of PAHs. In some selected studies, mice were co-housed. No dermal studies reported limiting either self-grooming or community grooming among mice. Consequently, a portion of the dermal dose may have been ingested by these animals.

Most PAH studies were completed prior to the availability of modern test methods and, consequently, there was no standard protocol to evaluate relative potency in the context of exposure duration, single versus multiple doses, frequency of dosing, age and strain of mice, selection of promotor chemical, etc. Given these limitations, it was not possible to determine how these variables may have affected the calculated relative potency. Most publications tested only a single dose of both the target PAH and BaP. Unfortunately, the methodology in publications meeting the a priori criteria was not consistently documented and most did not meet the dose–response criteria (i.e., high tumor response at the single or lowest dose tested), particularly for the common comparator, BaP.

The studies meeting the a priori criteria included both I/P and complete carcinogenicity studies. The US EPA [17] compared the PEFs calculated based on the results of I/P and complete carcinogenicity assays, as well as by route of exposure (Table 8-3 of that report). In many cases, the difference between the PEFs obtained from intraperitoneal administration and dermal exposure was larger than between I/P and complete carcinogenicity studies. It should also be noted that I/P studies reported tumor incidences based on papillomas, while longer-duration carcinogenicity studies reported data for both papillomas and carcinomas (Supplemental Table S1).

All PEFs in the current study were based on skin-painting experiments in mice, with most using acetone as the solvent. Unfortunately, potential effects of solvents on absorption, metabolism, and resulting tumor response of the target PAHs and BaP are largely unknown and/or not readily quantifiable. Kontir et al. [60] found all solvent vehicles studied inhibited benzo[a]pyrene hydroxylase in rabbit lung microsomes in a concentration-dependent manner, and that the magnitudes and types of PAH metabolites formed were highly dependent upon the specific solvent used as the vehicle.

Absence of datasets meeting our quantitative dose–response criteria for a particular PAH does not necessarily mean that qualitative potency data were lacking. For example, nine DBalP dermal carcinogenicity datasets from six publications that met the a priori criteria were identified (Table 5), and some included multiple doses of both DBalP and BaP. However, no DBalP studies met the dose–response criteria, as the tumor response was greater than 50% for DBalP or BaP at the only dose tested or the lowest nonzero dose. However, qualitative comparisons of potency were possible. For example, LaVoie et al. [36] tested four doses of DBalP and three doses of BaP in an I/P assay, and found that 1 nmol of DBalP resulted in a 95% response, but there was no statistically significant response at up to 25 nmol BaP, the highest dose tested. In a single-dose study with no promotion, Cavalieri et al. [30] found that 100 nmol DBalP induced papillomas and squamous cell carcinomas in 29% of mice, while 100 nmol BaP induced papillomas in 4% of mice tested (Table 4, footnote 1). These results were consistent with the published DBalP PEFs of 10−100 relative to BaP (see Table 2). In the literature, DBalP is considered the most potent PAH to date, but despite urging by scientists who have published papers on its toxicity (e.g. [54]), there is relatively limited information on its presence in environmental media.

As listed in Table 1 and Table 2, the values of published PEFs for the selected target PAHs ranged over several orders of magnitude and were typically based on qualitative estimation from experiments using a variety of routes of exposure, not all of which were environmentally relevant. PEFs calculated for BbF and CPP were within an order of magnitude or less of the PEFs reported in Table 1 and Table 2. Three experimental datasets met the criteria for calculating a quantitative PEF for CPP (two complete carcinogenicity studies and one I/P study) but there was no clear difference in potency by assay type. The CPP dataset was considered the best for estimating PEFs, with multiple experiments that met the criteria and assessed responses at multiple doses, but CPP is not included among the 16 PAHs routinely analyzed in environmental media or food (see review by [20]). Finally, although we calculated a PEF for BbF, the analysis was based on only a single experiment.

In summary, the confidence in, and overall utility of, the derived PEFs are limited by numerous uncertainties and overall poor quality of the datasets, the small number of PAHs for which PEFs can be derived, and the overall lack of PEFs for PAHs routinely included in environmental risk assessments.

### 4.2. Underlying Assumptions and Limitations of the Relative Potency Approach

The first key assumption of the PEF approach was that all PAHs act in a manner toxicologically similar to BaP [17]. While the SAB [10] and several published studies [45,61] have questioned this premise or noted concerns about the underlying justification, especially given the structural diversity of PAHs [62], they nonetheless concluded there was “adequate practical justification” for this approach in the near term in the absence of a good alternative. Labib et al. [61] demonstrated that in adult male Muta^TM^Mouse gavaged for 28 days with one of seven different PAHs, the induced tissue-specific perturbations of signaling pathways related to carcinogenesis were distinct from those produced by BaP. Tilton et al. [45] provided compelling transcriptomic evidence that BaP and DBalP (considered at least 10-fold more potent than BaP) can induce tumor development in mouse skin in vivo by different and unique mechanisms. While PEFs may correlate well with DNA adduct formation, adduct formation does not correlate well with the incidence of tumors induced by DBalP, suggesting that non-genotoxic mechanisms may contribute to carcinogenesis [41,45]. Gaylor et al. [63] noted the use of BaP as an index chemical to estimate lung cancer potency of PAH mixtures may be inappropriate because the lung is relatively insensitive to BaP carcinogenicity following oral exposure.

The second key assumption of the US EPA’s [17] relative potency approach is that the toxicity of PAHs is additive and other kinds of interactions between individual PAHs in a mixture do not occur at the low levels of exposure typically encountered in the environment. Aside from difficulty in defining “typical low levels of exposure”, this assumption is problematic for two reasons. First, current analytical methods and PEF determinations account for only a subset of PAHs and related compounds in mixtures, with significant uncertainties regarding presence of additional carcinogens or promoters not measured and/or addressed in the relative potency scheme [11]. Second, based on a variety of endpoints, including P450 induction, DNA adducts and Ah receptor binding, interactions between individual PAHs in a mixture may result not just in additive effects, but also synergistic or antagonistic effects in an unpredictable manner [64,65,66,67,68], although some studies reported no interactions [62,69,70]. Bauer et al. [71] provided some evidence that low molecular weight (LMW) PAHs, which are not generally considered carcinogenic, can enhance the carcinogenicity of BaP. Importantly, these PAHs (e.g., fluoranthene) are frequently the most abundant PAHs in complex mixtures encountered in environmental and occupational exposure scenarios. However, in another study that looked across the literature at PAH interactive genotoxicity (albeit based on bacterial mutagenicity), White [72] concluded that mutagenic risk posed by mixtures of PAHs could be reasonably estimated using additivity of the individual mixture components, even though small deviations from additivity were observed. Price et al. [73] summarized a number of studies that indicated that historical approaches involving in vivo studies with limited numbers of animals result in dose–response data that lack the statistical power to differentiate additivity from synergy or antagonism, especially when the chemicals in a mixture had non-linear dose–response relationships. The CCME [11] noted the PEF approach tended to underestimate the potency of coal tar and creosote mixtures typically found at contaminated sites. They therefore adopted a three-fold safety factor when using a relative potency approach to calculate the toxicity of creosote or coal tar mixtures; however, few other jurisdictions have applied a similar safety factor for PAH mixtures, and at least one recent study [18] found only modest deviations from additivity of priority PAHs when assessing the toxicity of two coal tars and one coal-tar-based pavement sealant.

In summary, data regarding the key assumptions of PEFs for assessing carcinogenic risk of PAHs in complex mixtures do not support the contentions that they function through a common mechanism of action. Indeed, several recent studies demonstrate that PAHs can act through unique mechanisms potentially contributing to cancer outcomes in a non-additive manner.

### 4.3. Next Steps

#### 4.3.1. Beyond the 16 Priority PAHs: Characterizing Complex PAH Mixtures, and Sources and Fate of PAHs in the Environment

Polycyclic aromatic compounds (PACs) are a large class of contaminants and include unsubstituted polycyclic aromatic hydrocarbons (PAHs) that only contain carbon and hydrogen, alkylated derivatives of PAHs (alk-PAHs), and heterocyclic aromatic compounds (containing N, O, or S-atoms). In the 1970s, 16 unsubstituted PAHs were identified by the US EPA as priority pollutants, and these pollutants in turn played an exceptional large role in the development of analytical methods [74,75], and in the associated environmental and analytical sciences in the last decades [20]. It has long been recognized that the focus on the 16 US EPA priority PAHs has limited the impetus to describe other PAHs in mixtures either analytically or from a toxicological/carcinogenic perspective [4,11,20]. Additionally, there has been failure to consider some highly toxic high molecular weight (HMW) PAHs, (e.g., DbalP) [20]; disregarding isomers of the priority PAHs may have interfered with laboratory measurements (e.g., chrysene, benz[a]anthracene, and benzofluoranthene [74]; b- and k- benzofluoranthene [11,74]. 

Combustion-generated PAH particles are not only composed of unsubstituted PAHs, but also include polar PAHs that include HMW PAHs, alkylated PAHs, and PAHs compounds containing heteroatoms (i.e., nitrogen, sulfur or oxygen atoms in an aromatic ring) and other substituted PACs such as amino-PAHs, cyano-PAHs, etc. [20,76,77,78]. Both nitro- and oxy-PAHs are products of incomplete combustion, while alkylated PAHs, are most commonly found with petrogenic PAHs.There is increasing evidence that toxicity, persistence, and mobility of polar PAHs in the environment differ from those of non-polar hydrophobic PAHs, because polar PAHs tend to be more reactive (e.g. [76,79]). Polar PAHs containing heteroatoms have shown toxicity similar to or greater than that of non-polar PAHs (e.g. [79,80,81,82]). Due to their unique hydrophobic nature, low water solubility and low vapor pressure (as reviewed in US EPA [83]), unsubstituted PAHs, including the 16 PAHs prioritized by the US EPA, tend to bind strongly to soil organic matter, thus reducing their mobility and bioavailability [84,85]. Weathered (aged or degraded) non-polar PAH mixtures in the environment are frequently composed of higher concentrations of HMW PAHs due to the progressive removal of LMW PAHs through photo-oxidation, leaching, volatilization and biodegradation, etc. ([86] cited in [87]). Most organic carbon in soil and sediment contains black carbon materials (e.g., soot, tar, and char) that primarily consist of slowly desorbing and irreversibly bound non-polar PAHs; other types of organic carbon, such as pitch, contain a mixture of rapidly desorbing, slowly desorbing, and irreversibly bound PAHs, depending on the production process and extent of weathering [85].

Based on the above considerations, characterization of PAH mixtures in their original and weathered forms, along with improved understanding, monitoring, and mapping of the fate of PAHs, will enable prioritization of the PAH mixtures most relevant to human health.

#### 4.3.2. Bioavailability

While some airborne or soil particulates may contain desorbable PAHs, non-polar PAHs can become irreversibly sequestered in carbon over time, and measurement of the total, rather than the bioaccessible/bioavailable PAH content in surface soils and sediments may lead to overestimation of potential exposure and risk [85,87].

In vitro bioaccessibility assays are often used as surrogate measures of bioavailability, but for hydrophobic contaminants, reported correlations between in vitro bioaccessibility and in vivo bioavailability have generally been poor [88]. Due to their static nature, in vitro assays often fail to provide sufficient sorption capacity, while newer bioaccessibility assays using sorption sinks can potentially better mimic dynamic intestinal uptake for hydrophobic organics such as PAHs from soil [88]. However, it is possible that neither the current gastrointestinal nor lung in vitro bioaccessibility assays provide sufficient sorption capacity for hydrophobic PAHs, which may result in underestimation of risk; therefore, it is essential that in vitro bioaccessiblity results be verified in vivo [88]. Future research addressing PAH bioavailability should not be restricted to unsubstituted (non-polar) PAHs, but should also examine the more reactive, albeit more complicated, polar PAHs.

Most studies of PAH bioavailability have focused on oral, and to a much lesser extent, dermal routes of exposure. Despite the greater exposure of humans to PAHs adsorbed to or contained within airborne particulate PAHs, the relationships between PAH bioaccessibility in PM_2.5_ (particulate matter with diameter less than 2.5 microns or µm) and adverse effects arising from exposure to particulate-associated PAHs have yet to be investigated [89].

#### 4.3.3. Relevant Indicator Compounds in PAH Mixtures

The limitations of current PAH mixture toxicity and the PEF approach, and the need for additional and more potent PAH indicator compounds is well recognized [4]. Richter-Brockmann and Achten [90] analyzed 59 PAHs in samples from petrogenic and pyrogenic sources, as well as samples from mixed environmental matrices. Using published PEFs for 24 PAHs, they found that non-EPA-priority PAHs made up 69.3–95.1% of the estimated potency. Standardized laboratory protocols similar to the EPA’s 16 PAH protocol are urgently needed for a much larger suite of polar and non-polar PAHs to allow routine measurement of their presence in various media and tissues.

#### 4.3.4. Alternative Toxicological Methods

Alternative high-throughput in vitro or in silico testing methods may allow for more rapid characterization of PAHs and PAH mixtures than is currently possible using in vivo assays, and can reduce or eliminate the use of laboratory animals (and potentially the cost of toxicity testing) in support of risk assessments of PAH mixtures.

One such area of active research is analysis of “omics” data from sub-chronic or short-term studies (e.g. [91]); however, most high-throughput in vitro test systems used to evaluate “omics” data currently have minimal or no metabolic capability and cannot capture communication between organs, although “organ on a chip” technology is developing rapidly. Considerable additional development work is needed before data from such alternative methods can be used to directly estimate potency of PAH mixtures. Testing strategies combining different types of assays, for example using in vitro or in silico analyses to address some issues, supplemented by targeted in vivo studies for validation of the results, may overcome some of the limitations of individual assays.

Alternative testing approaches are unlikely to identify all potential key events and their relationships to PAH toxicity or to divergence in cancer outcomes between exposure routes (i.e., oral, dermal, or inhalation). Instead, such approaches could focus on key elements of the toxicokinetics (e.g., bioaccessibility, key metabolic steps) and of the molecular initiating events (MIEs) and selected downstream events in a quantitative adverse outcome pathway. For example, although genotoxicity, DNA adducts, and AhR binding are not strong predictors of PAH carcinogenicity, a combination of these three endpoints might be predictive of comparative toxicity. Recently, Price et al. [73] proposed a source-to-outcome framework to examine the continuum of interactions between two chemicals focusing on adverse outcome (AOP;, i.e., toxicodynamic) and aggregate exposure (AEP) pathways, which may become applicable to more complex mixtures after further development.

## 5. Conclusions

Despite the known limitations of the PEF approach, it remains practical as a qualitative screening tool, and for exposure scenarios where the source and the composition of the PAH mixture have not been fully characterized.

Nonetheless, the key assumptions underlying PAH PEF derivations lack supporting data. The thinking that PAHs function through a common mechanism of action and in an additive manner has been challenged by recent studies demonstrating carcinogenic PAHs can act through multiple interacting mechanisms and that PAHs considered non-carcinogenic may nonetheless contribute to carcinogenesis. These limitations are particularly problematic for complex mixtures such as coal tar and creosote.

The analyses in the current paper showed that data suitable for calculating PEFs were almost wholly limited to those obtained from dermal exposure assays, and long-term oral or inhalation cancer studies are lacking for most individual PAH compounds other than BaP. In addition, no human epidemiological studies specifically addressing cancer risks resulting from oral exposure to PAHs were located; oral and dermal exposures in the available occupational studies were likely confounded by additional inhalation exposure. The abundance of rodent dermal PAH exposure studies may reflect the ease of administration compared to the oral or inhalation routes. However, dermal and oral bioavailability of PAHs may differ (e.g., due to first-pass metabolism following oral exposure), and apparent potency may vary depending on the solvent/vehicle used.

However, completion of multiple new animal studies meeting the study selection criteria recommended by the SAB [10] to derive exposure-route-specific PEFs for each PAH of interest is unlikely to be feasible in light of the costs, time, and number of animals required; such an approach would also be contrary to the current move away from animal testing. Further, the US EPA recently announced complete elimination of all testing in mammals by 2035 [92].

Finally, this analyses also demonstrated that there are currently insufficient data to develop new high-quality quantitative PEFs to replace the semi-quantitative PEFs currently available. Recently published reviews and experimental studies support future replacement of the PEF approach with an interdisciplinary mixtures approach. This shift will require improving our understanding of the fate and transport of selected PAH mixtures, deriving additional bioavailability data, expanding chemical analytical capabilities, and incorporating 21st century risk assessment methodologies (including in vitro and in silico techniques). In the interim, associated uncertainties must be explicitly addressed when relative potency is used for human health risk assessment of PAH-contaminated media.

## Figures and Tables

**Table 2:** Potency Factors of Additional Polycyclic Aromatic Hydrocarbon (PAH) Examined in This Study.

Carcinogenic Polycyclic Aromatic Hydrocarbon (PAH)	Short Form	CAS Number	IARC (2010) Class ^1^	IRIS US EPA RPF (2010) ^2^	PEF ^2^ Published
Benzo[c]fluorene	BcFE	205-12-9	3	20	20 ^3^
Benzo[c]phenanthrene	BcPh	195-19-7	2B	-	-
Cyclopenta[c,d]pyrene	CPP	27208-37-3	2A	0.4	0.1 ^4^
Dibenz[a,c]anthracene	DBacA	215-58-7	3	4	0.4–50 ^3^
Dibenzo[a,e]fluoranthene	DBaeF	5385-75-1	3	0.9	0.9 ^3^
Dibenzo[a,e]pyrene	DBaeP	192-65-4	3	0.4	1 ^3, 5^
Dibenzo[a,h]pyrene	DBahP	189-64-0	2B	0.9	10 ^3, 5^
Dibenzo[a,i]pyrene	DBaiP	189-55-9	2B	0.6	10 ^3, 5^
Dibenzo[a,l]pyrene	DBalP	191-30-0	2A	30	10 ^3, 5^, 100 ^4^
Alkylated PAHs					
5-Methylchrysene	5-MeC	3697-24-3	2B	-	1.0 ^4^

^1^ IARC Classification (2010) [1] defined in Table 1. ^2^ Draft IRIS (US EPA 2010) [17] asks to neither cite nor quote. Potency only provided for comparative purposes. ^3^ Summary of PEFs compiled by Andersson and Achten (2015) [20]. ^4^ OEHHA (2019) [21]. ^5^ EFSA (2008) [22].

**Table 3:** Criteria Used to Identify Target PAHs and Studies for Inclusion in PEF Assessment.

Evaluation Step	Criteria
Target PAH selection	8 Common PAHs (Table 1) Development of a relative potency approach for carcinogenic PAHs used for soil quality guidelines (CCME, 2010a, b) [11].
	10 Candidate PAHs (Table 2) Required for all: Laboratory standards/reference material are available, ^1^ The published PEF/RPF for the candidate PAH was usually greater than or equal to the potency of BaP, and/or The carcinogenic PAH candidate has been evaluated by IARC, with particular interest in those classified as 2B (possibly carcinogenic to humans) or higher.
Literature search for relevant studies	Broad search of scientific literature to identify animal bioassay studies that evaluated the carcinogenic potential and potency of 18 target PAHs concurrently with BaP, or another comparator chemical other than BaP.
A priori study inclusion	Inclusion of tumor incidence data for at least one of the target PAHs and BaP (IRIS, US EPA, 2010) [17]. Provision of tumor data from PAH administration by an environmentally route of exposure (oral, inhalation, or dermal) (SAB, US EPA, 2011) [10].
Dose–response inclusion	Preference for studies that examined multiple doses of BaP and multiple doses of the target PAH (SAB, US EPA, 2011) [10]. For those studies testing only a single dose of BaP and/or the target PAH, tumor response was ≤50% (SAB, US EPA, 2011) [10]. For multi-dose data sets, the response of lowest non-zero dose was ≤50% (this study).

^1^ Laboratory standards/reference material are necessary for development of analytical detection methods for PAHs in environmental media.

**Table 4:** List of References Selected by *A Priori* Criteria with Number of Studies, Type of Studies, and Target PAHs Examined.

PAH Studies	Target PAHs	Carcinogenicity (Complete or Initiation/Promotion)	Total No. Target PAH Studies in Paper
Barry et al., 1935 [26]	Benzo[c]phenanthrene	C	1
Cavalieri et al., 1977 [27]	Benz[a]anthracene	C	2
	Dibenzo[a,h]pyrene	C	
Cavalieri et al., 1981 [28]	Cyclopenta[c,d]pyrene	I/P, C	2
Cavalieri et al., 1983 [29]	Cyclopenta[c,d]pyrene	C	1
Cavalieri et al., 1991 ^1^ [30]	Dibenzo[a,l]pyrene	I/P	1
El-Bayoumy et al., 1982 [31]	Chrysene	I/P	1
Habs et al., 1980 [32]	Benzo[b]fluoranthene	C	5
	Benzo[j]fluoranthene	C	
	Benzo[k]fluoranthene	C	
	Indeno[1,2,3-cd]pyrene	C	
	Cyclopenta[c,d]pyrene	C	
Hecht et al., 1974 [33]	Chrysene	I\P	4
	5-Methylchrysene	I\P, I\P, C	
Higginbotham et al., 1993 [34]	Dibenzo[a,l]pyrene	I\P, C	2
Hoffmann and Wynder, 1966 ^2^ [35]	Benzo[g,h,i]perylene	I\P, C	12
	Indeno[1,2,3-cd]pyrene	I\P, C	
	Dibenzo[a,e]pyrene	I\P, C	
	Dibenzo[a,h]pyrene	I\P, C	
	Dibenzo[a,i]pyrene	I\P, C	
	Dibenzo[a,e]fluoranthene ^2^	I\P, C	
LaVoie et al., 1993 ^3^ [36]	Dibenzo[a,l]pyrene	I\P	1
LaVoie et al., 1982 [37]	Benzo[b]fluoranthene,	I\P	3
	Benzo[j]fluoranthene	I\P	
	Benzo[k]fluoranthene	I\P	
Raveh et al., 1982 [38]	Cyclopenta[c,d]pyrene	I\P	1
Rice et al., 1985 [39]	Chrysene	I\P	1
Rice et al., 1988 [40]	Chrysene	I\P	1
Siddens et al., 2012 ^3^ [41]	Dibenzo[a,l]pyrene	I\P	1
Siddens et al., 2015 ^3^ [42]	Dibenzo[a,l]pyrene	I\P	1
Slaga et al., 1978 [43]	Benz[a]anthracene	I\P	1
Slaga et al., 1980 [44]	Chrysene	I\P	3
	Dibenz[a,c]anthracene	I\P	
	Dibenz[a,h]anthracene	I\P	
Tilton et al., 2015 ^3^ [45]	Dibenzo[a,l]pyrene	I\P	1
Weyand et al., 1992 [46]	Benzo[j]fluoranthene	I\P	1
Weyand et al., 2004 [47]	Benzo[c]fluorene	C (oral route)	1
Wood et al., 1980 [48]	Cyclopenta[c,d]pyrene	I\P	1
Total No Studies			48

^1^ Third Initiation experiment of single dose of 100 nmol of BaP and DBalP without promotion not included. ^2^ Dibenzo[a,e]fluoranthene changed from Dibenzo[a,l]pyrene from Hoffman and Wynder (1966) [35] as per IRIS (US EPA, 2010) [17]; see text for further information. ^3^ References not included in IRIS (US EPA, 2010) [17].

**Table 5:** Target PAHs/Studies That Met A Priori Criteria but Did Not Meet Dose–response Criteria.

Chemical Name	Studies for Each Chemical That Met a Priori Criteria	Number of Doses (Target PAH/BaP)	Reason for Dose–Response Evaluation Failure ^1^			
			If Single Dose, Tumor Response for Target PAH and BaP ≤ 50%		If Multi-Dose, Lowest Dose Had Tumor Response for Target PAH and BaP ≤ 50%	
			PAH	%	PAH	%
Benz[a]anthracene	Cavalieri et al., 1977 [27]	1/1	No BaP	79		
	Slaga et al., 1978 [43]	1/1	No both	57/92		
Benzo[b]fluoranthene	LaVoie et al., 1982 [37]	3/1	No BaP	85		
Benzo[j]fluoranthene	LaVoie et al., 1982 [37]	3/1	No BaP	85		
	Weyand et al., 1992 [46]	3/1	No BaP	100		
Benzo[k]fluoranthene	LaVoie et al., 1982 [37]	3/1	No BaP	85		
Benzo[c]fluorene	Weyand et al., 2004 [47]	2/1	No BaP	77		
Benzo[g,h,i]perylene	Hoffmann & Wynder 1966 [35]	1/1	No BaP	80		
	Hoffmann & Wynder 1966 [35]	2/2			No BaP	85
Benzo[c]phenanthrene	Barry et al., 1935 ^2^ [26]	1/1	No BaP			
Chrysene	Hecht et al., 1974 [33]	1/1	No CH	61		
	Hecht et al., 1974 ^3^ [33]	1/1	No BaP	65		
	Slaga et al., 1980 [44]	1/1	No both	73/67		
	El-Bayoumy et al., 1982 [31]	1/1	No both	100/90		
	Rice et al., 1985 [39]	1/1	No both	92/96		
	Rice et al., 1988 [40]	3/1	No BaP	89		
Cyclopenta[c,d]pyrene	Wood et al., 1980 ^4^ [48]	2/2			No BaP	21/68
Dibenz[a,c]anthracene	Slaga et al., 1980 [44]	1/1	No BaP	67		
Dibenz[a,h]anthracene	Slaga et al., 1980 [44]	1/1	No BaP	67		
Dibenzo[a,e]fluoranthene	Hoffman & Wynder, 1966 [35]	1/1	No BaP	93		
	Hoffman & Wynder, 1966 [35]	2/2			No BaP	85
Dibenzo[a,e]pyrene	Hoffman & Wynder, 1966 [35]	1/1	No BaP	85		
	Hoffman & Wynder, 1966 [35]	2/2			No BaP	93
Dibenzo[a,h]pyrene	Cavalieri et al., 1977 [27]	1/1	No both	90/79		
	Hoffman & Wynder, 1966 [35]	1/1	No BaP	85		
	Hoffman & Wynder, 1966 [35]	2/2			No BaP	93
Dibenzo[a,i]pyrene	Hoffman & Wynder, 1966 [35]	1/1	No BaP	85		
	Hoffman & Wynder, 1966 [35]	2/2			No BaP	93
Dibenzo[a,l]pyrene	Cavalieri et al., 1991 [30]	3/3			No both	100/91
	Cavalieri et al., 1991 [30]	3/3			No Both	83/92
	Higginbotham et al., 1993 ^5^ [34]	2/1	No BaP			
	Higginbotham et al., 1993 ^6^ [34]	3/3			No BaP	
	LaVoie et al., 1993 [36]	4/3			No DBalP	95
	Siddens et al., 2015 [42]	1/1	No both	100/89		
	Siddens et al., 2012 [41]	1/1	No both	100/78		
	Tilton et al., 2015 [45]	1/1	No DBalP	88		
Indeno[1,2,3-cd]pyrene	Hoffmann and Wynder, 1966 ^7^ [35]	1/1	No both	85/80		
	Hoffmann and Wynder, 1966 ^7,8^ [35]	1/2			No BaP	85
5-Methylchrysene	Hecht et al., 1974 [33]	1/1	No 5-MeC	94		
	Hecht et al., 1974 [33]	2/1	No BaP	95	No 5-MeC	100
	Hecht et al., 1974 ^9^ [33]	1/1	No Both	100		

^1^ To meet dose–response criteria, the following must be true for *both* BaP and the target PAH: tumor response was ≤50% at the single or lowest (in multi-dose studies) tested dose. Failure of either target PAH or BaP to meet minimal dose–response is identified in the appropriate single or multiple dose column depending on the number of dose levels tested. Failure also occurred when there was no dose–response or there was a non-positive result at either the single dose tested or at the highest dose of multiple dose studies. Where two percentages are listed, the first percentage refers to the target PAH and the second to BaP. ^2^ BcPh—incidences of animals with tumors were not provided; 1 dose of BaP, response incidence was unclear. ^3^ By end of complete carcinogenicity study, BaP had 65% mortality and CH had 45% with 65 and 20 % incidence, respectively. ^4^ CPP—maximum response was 21% and study authors considered this result non-positive. ^5^ DBalP—BaP was “inactive” at the one dose tested; primary BaP and control response data not provided. ^6^ DBalP—0 tumor bearing animals at all 3 doses of BaP. ^7^ IP—The number of mice with papillomas and with epitheliomas was provided; it is not clear whether double-counting was avoided. ^8^ IP—Table 2 in Hoffman and Wynder (1966) [35] cites a 1961 paper with better dose–response, but the full citation was not provided, and the original paper could not be located. ^9^ 5-MeC—100% mortality by week 35 and 100% tumor incidence by week 25 in a 72-week study.

**Table 6:** Target PAHs/Studies that met Dose–Response Criteria.

Chemical Name	Studies for Each Chemical That Met a Priori Criteria and Dose Response Criteria	Number of Doses (PAH/BaP)	Dose–Response Comments
Benzo[b]fluoranthene	Habs et al., 1980 ^1^ [32]	3/3	Good dose–response for BbF and BaP.
Benzo[j]fluoranthene	Habs et al., 1980 [32]	3/3	Minimal tumor response: only 5% response at the highest dose tested.
Benzo[k]fluoranthene	Habs et al., 1980 [32]	3/3	Minimal tumor response: only one mouse with tumor and at the lowest dose.
Indeno[1,2,3-cd]pyrene	Habs et al., 1980 [32]	3/3	Minimal tumor response: only one mouse with tumor and at the lowest dose.
Cyclopenta[c,d]pyrene	Cavalieri et al., 1981 [28]	3/1	Dose–response for control was 10%. Dose–response of CPP non-monotonic. ^2^
	Cavalieri et al., 1983 [29]	3/3	Good dose–response for CPP and BaP.
	Habs et al., 1980 [32]	3/3	Good dose–response for CPP and BaP.
	Raveh et al., 1982 [38]	5/3	Good dose–response for CPP; BaP response at lowest dose was 58%.

^1^ Bolded studies were used to calculate PEFs. ^2^ Non-monotonic: highest response was not at the highest dose.

**Table 7:** Summary of PEF Calculations from Dose Response Modelling.

Target PAH	Study	BMD for BaP	BMD for Target PAH	PEF	Study Type
BbF	Habs et al., 1980 [32]	1.10	4.59	0.24	Complete
CPP	Cavalieri et al., 1983 [29]	1.87	15.49	0.12	Complete
	Habs et al., 1980 [32]	1.10	36.57	0.03	Complete
	Raveh et al., 1982 [38]	2.38	40.72	0.06	I/P
	Average of CPP PEFs			0.07	

PEF = (BMD_10_ for BaP)/(BMD_10_ for target PAH.).

## Supplementary Materials

The following supporting information can be downloaded at: https://www.mdpi.com/article/10.3390/ijerph19159490/s1, Table S1: Parameters of Reviewed Studies Meeting a priori Criteria; Table S2: Summary of Study Design and Dose-Response Information for Studies that met Dose Response Criteria in Table 6; Table S3: Summary of BMD Modeling Results.

## Abbreviations

5-MeC—5-Methylchrysene; AEP—aggregate exposure pathway; AOP—adverse outcome pathway; BaA—Benz[a]anthracene; BaP—Benzo[a]pyrene; BbF—Benzo[b]fluoranthene; BcFE—Benzo[c]fluorene; BcPh—Benzo[c]phenanthrene; BghiP—Benzo[g,h,i]perylene; BjF—Benzo[j]fluoranthene; BkF—Benzo[k]fluoranthene; BMA—Bayesian model averaging; BMD—benchmark dose; BMD10—the dose estimated to correspond to a 10% response; BMDL—lower 95% confidence limit of the benchmark dose; BMR—benchmark response; CCME—Canadian Council of Ministers of the Environment; CH—Chrysene; CPP—Cyclopenta[c,d]pyrene; DBacA—Dibenz[a,c]anthracene; DBaeF—Dibenzo[a,e]fluoranthene; DBaeP—Dibenzo[a,e]pyrene; DBahA—Dibenz[a,h]anthracene; DBahP—Dibenzo[a,h]pyrene; DBaiP—Dibenzo[a,i]pyrene; DBalP—Dibenzo[a,l]pyrene; EFSA—European Food Safety Authority; HMW—high molecular weight; IARC—International Agency for Research on Cancer; IP—Indeno[1,2,3-cd]pyrene; I/P—initiation/promotion; IPCS—International Programme on Chemical Safety; IRIS—Integrated Risk Information System; LMW—low molecular weight; MIE—molecular initiating event; NTP—National Toxicology Program; OEHHA—Office of Environmental Health Hazard Assessment; PAC—polycyclic aromatic compounds; PAH—polycyclic aromatic hydrocarbon; PEF—potency equivalence factor; PM 2.5—particulate matter with diameter less than 2.5 microns; RPF—relative potency factor; SAB—Science Advisory Board; SQG—Soil Quality Guidelines; TPA—tetradecanoyl phorbol acetate; US EPA—United States Environmental Protection Agency; WHO—World Health Organization.
