# Supplementary material for: The Long Goodbye: Finally Moving on from the Relative Potency Approach to a Mixtures Approach for Polycyclic Aromatic Hydrocarbons (PAHs)
Source: Int J Environ Res Public Health. 2022 Aug 2;19(15):9490. doi: 10.3390/ijerph19159490 (PMC9368405; doi:10.3390/ijerph19159490)

Table S1. Parameters of Reviewed Studies Meeting a priori Criteria (all were dermal studies in female mice)

INITIATION/PROMOTION STUDIES

Author	Age (weeks) and Strain of mice	Dosing Period & Frequency		Total Exposure Period (weeks)	Solvent	Purity (PAH/BaP) (%)	Types of Tumor
		Initiation (days)	Promotion (weeks)				
Cavalieri et al. 1981 [41]	9 week old Swiss	20 days, Subdoses every 2 days	40 weeks, TPA 2x/week	57	acetone	99.7/NR	Papilloma
Cavalieri et al. 1991 [39] (1st exper't)	8-week old SENCAR	Single dose	12 weeks, 1 week TPA, 2 week pause until TPA resumption TPA 2x/week	16	acetone	99.7/NR	Papilloma
Cavalieri et al. 1991 [39] (2nd exper't)	8-week old SENCAR	Single dose	24 weeks , 3 week pause until TPA resumption TPA 2x/week	25			
Cavalieri et al. 1991 [39] (3rd exper't)	8-week old SENCAR	Single dose	No promotion	27			
El-Bayoumy et al. 1982 [82]	4 – 5 week old Crl-CD-1	20 days Subdoses every two days	25 weeks, TPA 3x/week	45	acetone	>99/>99	Primarily squamous cell papilloma
Hecht et al. 1974 [33]	(age not specified) Crl-CD-1	20 days Subdoses every two days	20 weeks, after 10 days post-initiation TPA 3x/week	40	acetone	>99.9/NR	Papillomas, carcinomas
Higginbotham et al. 1993 [30]	8 week old SENCAR	Single dose	27 weeks, TPA 2x/week	28	acetone	>99/>99	Papilloma, squamous cell carcinomas
Hoffman and Wynder 1966 [35]	7-8 week old Ha/ICR/Mil Swiss	Single dose	Croton oil 6 months	24	dioxane	NR/93	Papilloma, epithelioma
LaVoie et al. 1982	4 – 5 week old Crl:CD-1	20 days Subdoses every two days	20 weeks, TPA 2x/week	23	acetone	Purchased [1]	Not specified

Author	Age (weeks) and Strain of mice	Dosing Period & Frequency		Total Exposure Period (weeks)	Solvent	Purity (PAH/BaP) (%)	Types of Tumor
		Initiation (days)	Promotion (weeks)				
LaVoie et al. 1993 [83]	4 – 5 week old CD-1	20 days Subdoses every two days	20 weeks, TPA 3x/week	23	acetone	>99/>99	Primarily squamous cell papilloma
Raveh et al. 1982 [42]	7-9 week old SENCAR (skin tumor sensitive)	Single dose	25 weeks, TPA 2x/week	25	NR	NR/NR	Papilloma
Rice et al. 1985 [84]	age not specified CD-1 mice	20 days Subdoses every two days	20 weeks, TPA 3x/week	23	acetone	>99/>99	Not specified
Rice et al. 1988 [85]	age not specified CD-1 mice	20 days Subdoses every two days	20 weeks, TPA 3x/week	23	acetone	>99/>99	Not specified
Siddens et al. 2012 [37]	7.5 weeks-old FVB/N inbred	Single dose	20 weeks after 2 weeks post-initiation, TPA 2x week	22	toluene	Purchased [1]	Papillomas, squamous cell carcinoma and carcinoma in situ
Siddens et al. 2015 [38]	7.5 weeks-old *Cyp 1b1* null	Single dose	20 weeks after 2 weeks post-initiation, TPA 2x week	22	toluene	Purchased [1]	Papilloma, squamous cell carcinoma
Slaga et al. 1978 [86]	7-9 week-old CD-1	Single dose	26 weeks, TPA 2 times/week	27	acetone	>99/>99	Papilloma
Slaga et al. 1980 [31]	7-9 week-old SENCAR	Single dose	15 weeks, TPA 2x/week	16	acetone	>99/>99	Papilloma
Tilton et al. 2015 [40]	7.5 week-old FVB/N inbred	Single dose	25 weeks, after 2 weeks post-initiation; TPA 2x week	27	toluene	Purchased [1]	Papillomas, squamous cell carcinoma and carcinoma in situ [2]
Weyand et al. 1992 [87]	7 – 8 week old Crl:CD-1	10 or 20 days, dose every other day	20 weeks, after 10 days post-initiation; TPA 3x week	21.4	acetone	>99/>99	Not specified
Wood et al. 1980 [32]	8-week old CD-1	Single dose	26 weeks, TPA 2x week	26	acetone	NR/>98%	Papilloma

[1] Not reported, but all stated they were purchased form commercial laboratories, so the assumption is that the purity is high.
[2] This was the only study that measured tumor incidence as the percent incidence for each tumor type.
NR = Not reported

Complete Carcinogenicity Studies

Author	Age (weeks) and Strain of mice	Target PAH Exposure	Protocol for Mice Dying during Experiment	Total Exposure Period	Solvent	Purity (PAH/BaP) (%)	Types of Tumor
Barry et al. 1935 [29]	Unspecified	2x week; 1 – 2+ years	Not specified	Until end of life	benzene	varying	Papilloma, epithelioma
Cavalieri et al. 1977 [81]	7 week-old random bred Swiss	30 weeks; 2x/week	Moribund or dead	Sacrificed survivors at 70 weeks	acetone	99/99	Papilloma, kerato-acanthoma, carcinoma
Cavalieri et al. 1981 [41]	9 week-old CD-1	30 weeks; 2x/week	Until 2 cm tumor or moribund	Sacrificed survivors at 57 weeks	acetone	99.7/NR	Papilloma
Cavalieri et al. 1983 [43]	9 week old Swiss	48 weeks; 2x /week	Until tumor size > 2 cm	Sacrificed survivors at 61 weeks	acetone	99.7/NR	Sum of papilloma, sebaceous gland adenomas, squamous cell carcinomas, basal cell carcinomas
Habs et al. 1980 [36]	10-week old NMRI	Life; 2x/week	Obvious tumor growth	Until end of life	acetone	>96/>96	Sum of Papilloma, carcinoma, sarcoma
Hecht et al. 1974 [33]	albino Ha/ICR/Mil Swiss	17 months, 3x/week	Not specified	Sacrificed survivors at 72 weeks. 5-MeCH 100% mortality at 35 weeks; BaP 65% mortality at week 70.	acetone	>99.9/NR	Papillomas, carcinomas
Higgenbotham et al. 1993 [30]	8 week-old Swiss	40 weeks; 2x/week	Until tumor size > 2 cm	48 weeks	acetone	>99/>99	Papilloma, squamous cell carcinomas
Hoffman and Wynder 1966	7-8 week-old Ha/ICR/Mil Swiss	12 months; 3x/week	Not specified	15 months	dioxane	NR/93	Papilloma, epithelioma

Table S2. Summary of Study Design and Dose-Response Information for Studies that met Dose Response Criteria in Table 6.

Study	Chemical	Dose Units	Dose of PAH	Dose Mass (µg/mouse) [1]	Number of animals with tumours	Number animals/ group	% tumour bearing animals	Tumour Type
**Cavalieri et al. 1981 [41]**	Solvent - acetone	33.4 µl	0	0	3	29	10	Mostly Papillomas.
	Benzo[a]pyrene	µmol	0.2	50.5	12	30	40	
	Cyclopenta[c,d]pyrene		0.2	45.3	3	30	1	
			0.6	135.8	9	30	31	
			1.8	407.3	6	30	21	

Study Type: Initiation/Promotion
<u>**Study Details**</u>
Exposure Duration: 9-week old female Swiss albino mice in groups of 30 were exposed dermally every other day for 20 days for a total of 10 subdoses in 33.4 µL of acetone. Promotion started after last treatment using TPA (12-O-tetradecanoylphorbol-13-acetate) twice weekly at a dose of 0.017 µmol in 33 µL of acetone for 40 weeks. Mice were killed when skin tumors were 2 cm in diameter or larger, and/or mice were moribund. Survivors were sacrificed after 57 experimental weeks.
Mortality: No information was provided on time to tumors and dose dependency. No information was provided on mortality of mice before end of experiment.
Purity: Not reported.
Note: 10% tumor incidence for acetone control is problematic

Study	Chemical	Dose Units	Dose of PAH	Dose Mass (µg/mouse) [1]	Number of animals with tumours	Number animals/ group	% tumour bearing animals	Tumour type
**Cavalieri et al. 1983 [43]**	Solvent - acetone	50 µL	0	0	0	29	0	Papilloma, sebaceous gland adenomas,
	Benzo[a]pyrene		2.2	0. 6	2	30	7	
		nmol	6.6	1.7	2	28	7	

Study	Chemical	Dose Units	Dose of PAH	Dose Mass (µg/mouse) [1]	Number of animals with tumours	Number animals/ group	% tumour bearing animals	Tumour Type
			20	5.1	17	30	57	and squamous cell carcinomas.
	Cyclopenta[c,d]pyrene		22.2	5.0	2	29	7	
		nmol	66.6	15.1	2	29	7	
			200	45.3	24	29	83	

Study Type: Complete Carcinogenicity

Study Details

Exposure Duration: 9-week old female Swiss mice were exposed dermally to CP twice weekly in acetone for 48 weeks. It is assumed sub-doses were applied in twice weekly application. Mice were killed when skin tumors were at least 2 cm in diameter and survivors sacrificed after 61 experimental weeks.

Note: Only tumor incidence data provided so number of animals with skin tumor incidence calculated from % tumor-bearing animals.

Mortality: No information was provided on time to tumors and dose dependency. No information on whether survival was statistically affected at any dose.

Purity: Available for CPP in another paper, but not reported for BaP.

Study	Chemical	Dose Units	Dose of PAH	Dose Mass (µg/mouse) [1]	Number of animals with tumours	Number animals/ group	% tumour bearing animals	Tumour type
**Habs et al. 1980 [36]**	Control - Acetone	20 µL	0	0	0	35	0	Type of tumors was not provided, but based on text, papillomas, sarcomas and carcinomas were mentioned for a few chemicals.
	Benzo[a]pyrene	µg/ mouse		1.7	8	34	24	
				2.8	24	35	69	
				4.6	22	36	61	
	Benzo[b]fluoranthene	µg/ mouse		3.4	2	38	5	
				5.6	5	34	15	
				9.2	20	37	54	
	Benzo[j]fluoranthene	µg/		3.4	1	38	3	

Study	Chemical	Dose Units	Dose of PAH	Dose Mass (µg/mouse) [1]	Number of animals with tumours	Number animals/ group	% tumour bearing animals	Tumour Type
		mouse						
				5.6	1	35	3	
				9.2	2	38	5	
	Benzo[k]fluoranthene	µg/ mouse		3.4	1	39	3	
				5.6	0	38	0	
				9.2	0	38	0	
	Cyclopenta[c,d]pyrene	µg/ mouse		1.7	0	34	0	
				6.8	0	35	0	
				27.2	3	38	8	

Study Type: Complete Carcinogenicity
<u>**Study Details**</u>
Exposure Duration: 10-week old NMRI Mice exposed 2x/week for life, until moribund or dead. The amount of acetone was not specified for controls. We assumed that each application were in subdoses of 0.02 ml acetone. Animals were housed 10 mice per cage. Animals at an advanced stage of macroscopically clearly infiltrative tumor growth were killed prior to their natural death, all the other animals were observed until their natural death. Authors also reported age-standardized tumour frequency; but did not specify how this was calculated.
Mortality: No information was provided on time to tumors and dose dependency, nor on whether survival was statistically affected at any dose.
Purity: All PAHs >96%

Study	Chemical	Dose Units	Dose of PAH	Dose Mass (µg/mouse) [1]	Number of animals with tumours	Number animals/ group	% tumour bearing animals	Tumour Type
**Raveh et al. 1982 [42]**	Control – chemical name not provided	NR[2]		NR	3	29	10	Papillomas
	Benzo[a]pyrene	µg/ mouse		10	17	29	59	

Study	Chemical	Dose Units	Dose of PAH	Dose Mass (µg/mouse) [1]	Number of animals with tumours	Number animals/ group	% tumour bearing animals	Tumour Type
				25	21	28	75	
				50	24	28	86	
				100	27	27	100	
				200	26	26	100	
	Cyclopenta(cd)pyrene	µg/ mouse		10	3	30	10	
				100	11	29	38	
				200	16	28	57	

Study Type: Initiation/Promotion

Study Details

Exposure Duration: 7-9-week old female SENCAR (skin tumor sensitive) mice in groups of 30 were exposed dermally in a single dose. Promotion began a week later with 2 µg TPA applied twice weekly for 25 weeks. Tumor incidence calculated from % tumor bearing animals but conversion to number of animals did not result in whole animal numbers for several groups. Adjustment of ± 1% resulted in appropriate numbers of animals. It was not clear why the estimated incidence does not correctly back-calculate to % tumor bearing animals. And the only possibility is incorrect rounding errors. Use of solvent for control animals not indicated.

Mortality: The number of papillomas per mouse at 25 weeks after initiation was dose dependent up to 200 /µg/ml in the case of CPP and up to 100 /µg/ml in the case of BaP. Not clear whether survival was statistically affected at any dose.

Purity: Not reported.

[1] Dose converted to µg/mouse from other units if authors did not provide units as µg/mouse

[2] NR = Not reported

Reference

Cavalieri, E.; Rogan, E.; Toth, B.; Munhall, A. Carcinogenicity of the environmental pollutants cyclopenteno-(cd) pyrene and cyclopentano(cd)pyrene in mouse skin. *Carcinogenesis* **1981,** *2* (4), pp. 277-281.

Cavalieri, E.; Munhall, A.; Rogan, E.; Salmasi, S.; Patil, K. Syncarcinogenic effect of the environmental pollutants cyclopenteno[cd]pyrene and benzo[a]pyrene in mouse skin. *Carcinogenesis* **1983,** *4* (4), pp. 393-397.

Habs, M.; Schmähl, D.; Misfeld, J. Local carcinogenicity of some environmentally relevant polycyclic aromatic hydrocarbons after lifelong topical application to mouse skin. *Arch Geschwulstforsch* **1980,** *50* (3), pp. 266-74.

Raveh, D.; Slaga, T. J.; Huberman, E. Cell-mediated mutagenesis and tumor-initiating activity of the ubiquitous polycyclic hydrocarbon, cyclopenta[c,d]pyrene. *Carcinogenesis* **1982,** *3* (7), pp. 763-766.

Table S3. Summary of BMD Modeling Results.

Habs et al., 1980. BaP (Including high dose)

Model Name	Posterior Probability	BMD	BMDL	BMDU	P-value	AIC	Scaled residual for dose group nearest the BMD	Scaled residual for control group
Dichotomous Hill	0.14	1.57	1.11	1.67	0.50	135.23	0.00	0.00
Gamma	0.06	0.48	0.02	1.05	0.01	142.34	0.00	0.00
Logistic	0.00	1.10	0.88	1.37	0.00	151.35	-0.24	-1.81
LogLogistic	0.14	0.67	0.13	1.15	0.01	141.59	0.00	0.00
LogProbit	0.05	0.73	0.15	1.21	0.01	141.61	0.00	0.00
Multistage-3	0.13	1.48	1.10	1.64	NA	136.79	0.00	0.00
Multistage-2	0.13	0.37	0.22	0.77	0.01	142.24	0.00	0.00
Multistage-1	0.13	0.42	0.34	0.54	0.03	140.37	0.00	0.00
Probit	0.00	1.06	0.85	1.33	0.00	150.29	-0.17	-1.63
Quantal-Linear	0.43	0.42	0.34	0.54	0.06	138.37	0.00	0.00
Weibull	0.05	0.44	0.04	0.93	0.01	142.36	0.00	0.00
BMA	N/A	0.58	0.33	1.12	-	-	-	-

Inadequate fit – and dichotomous Hill model is overparameterized, so this is just presented for context, no figure.

Habs et al., 1980. BaP (High dose dropped)

Model Name	Posterior Probability	BMD	BMDL	BMDU	P-value	AIC	Scaled residual for dose group nearest the BMD	Scaled residual for control group
Dichotomous Hill	0.05	1.31	0.84	1.61	NA	88.67	0.00	0.00
Gamma	0.02	1.31	0.78	1.61	1.00	84.67	0.00	0.00
Logistic	0.08	1.30	0.96	1.62	0.49	85.48	0.35	-0.57
LogLogistic	0.11	1.31	0.84	1.61	NA	86.67	0.00	0.00
LogProbit	0.12	1.35	0.90	1.62	NA	86.67	0.00	0.00
Multistage-3	-	-	-	-	-	-	-	-
Multistage-2	0.02	1.36	0.65	1.64	NA	86.67	0.00	0.00
Multistage-1	0.02	0.36	0.27	0.50	0.02	90.77	0.00	0.00
Probit	0.43	1.27	0.91	1.60	0.69	84.94	0.20	-0.33
Quantal-Linear	0.02	0.36	0.27	0.50	0.06	88.77	0.00	0.00
Weibull	0.14	1.24	0.72	1.59	NA	86.67	0.00	0.00
**BMA**	-	**1.10**	0.54	1.50	-	-	-	-

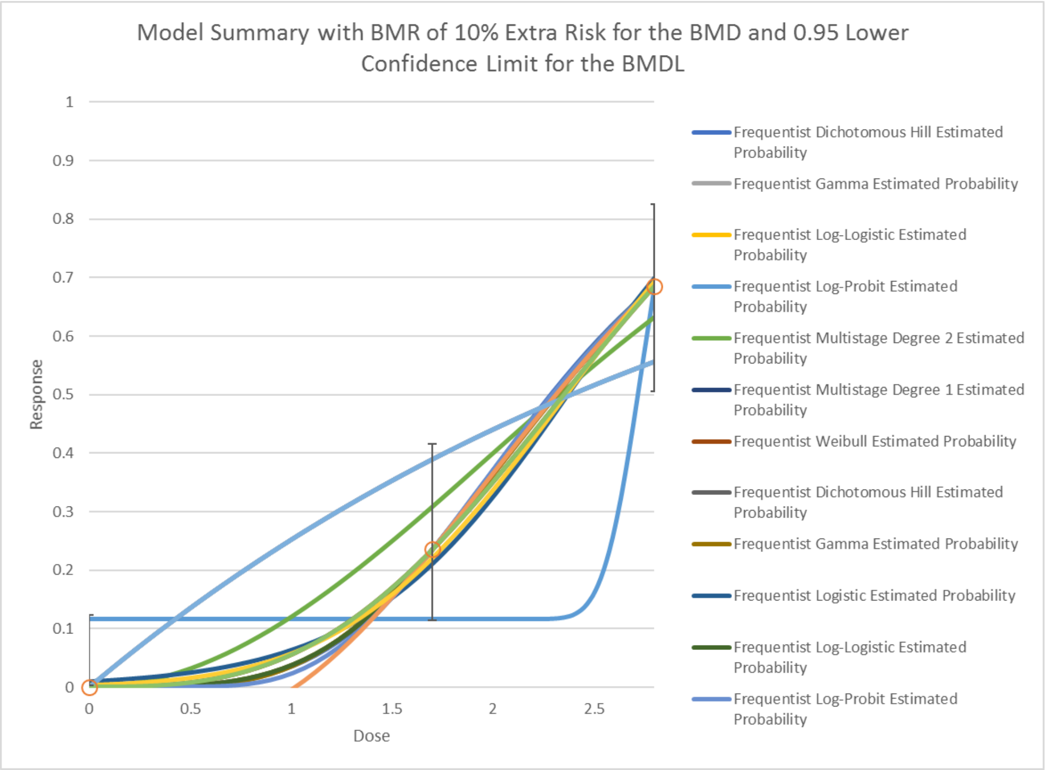

Habs et al., 1980. BbF

**Model Name**	**Posterior Probability**	**BMD**	**BMDL**	**BMDU**	**P-value**	**AIC**	**Scaled residual for dose group nearest the BMD**	**Scaled residual for control group**
Dichotomous Hill	0.06	4.59	3.50	5.49	0.52	101.52	-0.45	0.00
Gamma	0.02	4.54	3.45	5.38	0.79	99.58	-0.51	0.00
Logistic	0.08	5.03	4.09	5.93	0.86	99.68	0.11	-0.50
LogLogistic	0.12	4.59	3.50	5.49	0.52	101.52	-0.45	0.00
LogProbit	0.13	8.49	0.00	9.25	0.01	108.76	0.00	-1.57
Multistage-3	0.00	4.80	0.93	6.13	NA	103.11	0.00	0.00
Multistage-2	0.00	4.37	3.30	5.17	0.74	99.75	0.28	0.00
Multistage-1	0.00	2.12	1.56	2.97	0.01	109.55	-1.75	0.00
Probit	0.47	4.81	3.87	5.68	0.91	99.39	-0.11	-0.31
Quantal-Linear	0.00	2.12	1.56	2.96	0.02	107.55	-1.75	0.00
Weibull	0.12	4.62	3.44	5.58	0.91	99.31	-0.32	0.00
**BMA**	-	**4.59**	3.51	5.70	-	-	-	-

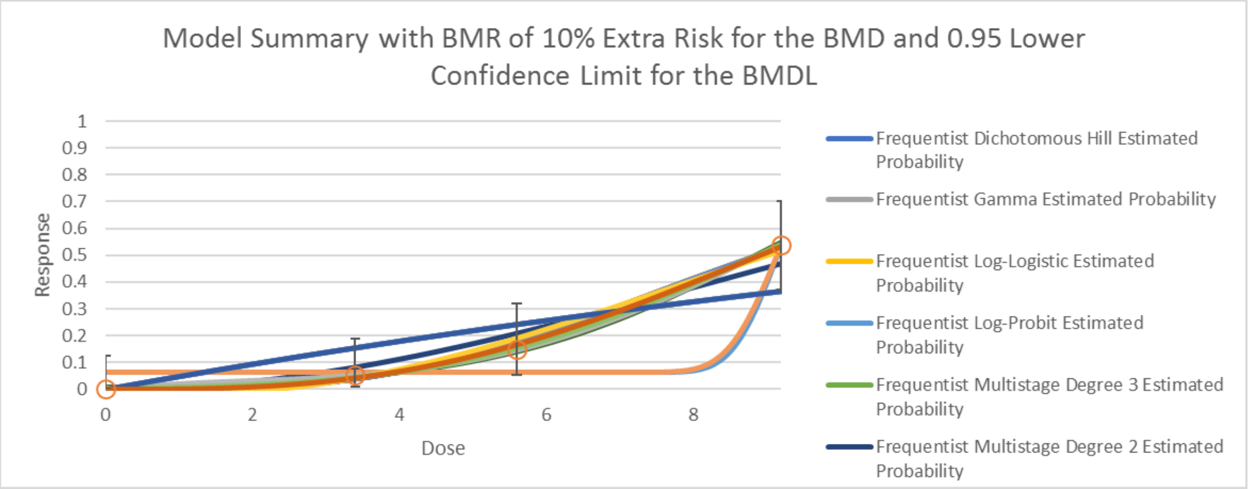

Habs et al., 1980. CPP

Model Name	Posterior Probability	BMD	BMDL	BMDU	P-value	AIC	Scaled residual for dose group nearest the BMD	Scaled residual for control group
Dichotomous Hill	0.17	27.96	8.23	53.42	1.00	24.99	0.00	0.00
Gamma	0.02	28.16	21.24	Infinity	1.00	24.99	0.00	0.00
Logistic	0.13	27.65	24.33	41.75	1.00	22.99	0.00	0.00
LogLogistic	0.00	27.59	21.32	Infinity	1.00	22.99	0.00	0.00
LogProbit	0.02	NA	0.00	Infinity	0.00	35.08	2.48	-0.87
Multistage-3	0.00	27.26	8.14	28.00	NA	28.99	0.00	0.00
Multistage-2	0.00	30.26	21.42	Infinity	1.00	24.99	0.00	0.00
Multistage-1	0.00	45.24	19.90	Infinity	0.64	26.56	0.45	0.00
Probit	0.65	27.81	23.58	44.89	1.00	24.99	0.00	0.00
Quantal-Linear	0.00	45.24	19.91	Infinity	0.83	24.56	0.45	0.00
Weibull	0.00	27.58	21.51	Infinity	1.00	22.99	0.00	0.00
**BMA**		**36.57**	23.05	Infinity	-	-	-	-

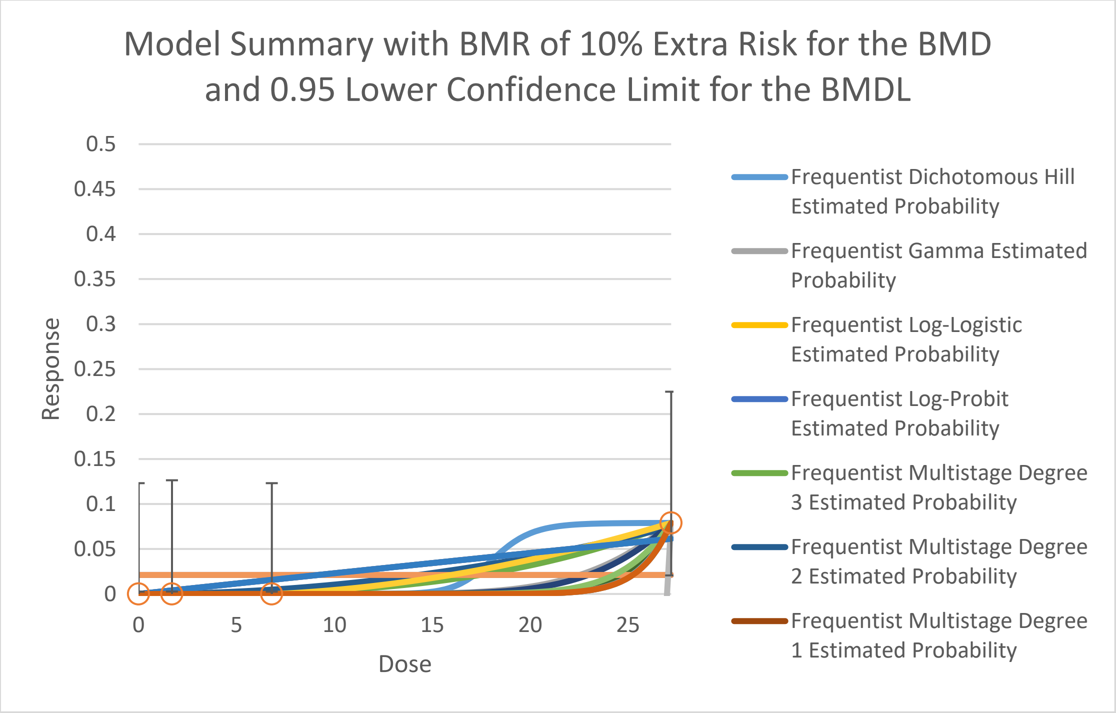

Cavalieri et al. 1983. BaP

Model Name	Posterior Probability	BMD	BMDL	BMDU	P-value	AIC	Scaled residual for dose group nearest the BMD	Scaled residual for control group
Dichotomous Hill	0.08	2.14	0.71	4.72	NA	80.82	-0.17	-0.94
Gamma	0.06	1.22	0.63	1.81	0.26	76.90	-1.19	0.00
Logistic	0.11	2.22	1.70	2.81	0.45	76.24	-0.19	-0.82
LogLogistic	0.16	2.14	0.71	4.57	0.15	78.82	-0.17	-0.94
LogProbit	0.11	2.19	0.76	4.85	0.16	78.92	-0.02	-1.00
Multistage-3	0.02	3.23	0.28	4.41	NA	78.16	0.00	0.00
Multistage-2	0.02	1.34	0.72	2.69	0.16	78.05	-0.96	0.00
Multistage-1	0.02	0.83	0.59	1.22	0.16	78.43	-0.03	0.00
Probit	0.28	2.00	1.55	2.54	0.43	76.19	-0.35	-0.74
Quantal-Linear	0.05	0.83	0.59	1.22	0.16	78.43	-0.03	0.00
Weibull	0.15	1.95	0.66	4.58	0.14	78.72	-0.42	-0.83
**BMA**	-	**1.87**	0.91	2.84	-	-	-	-

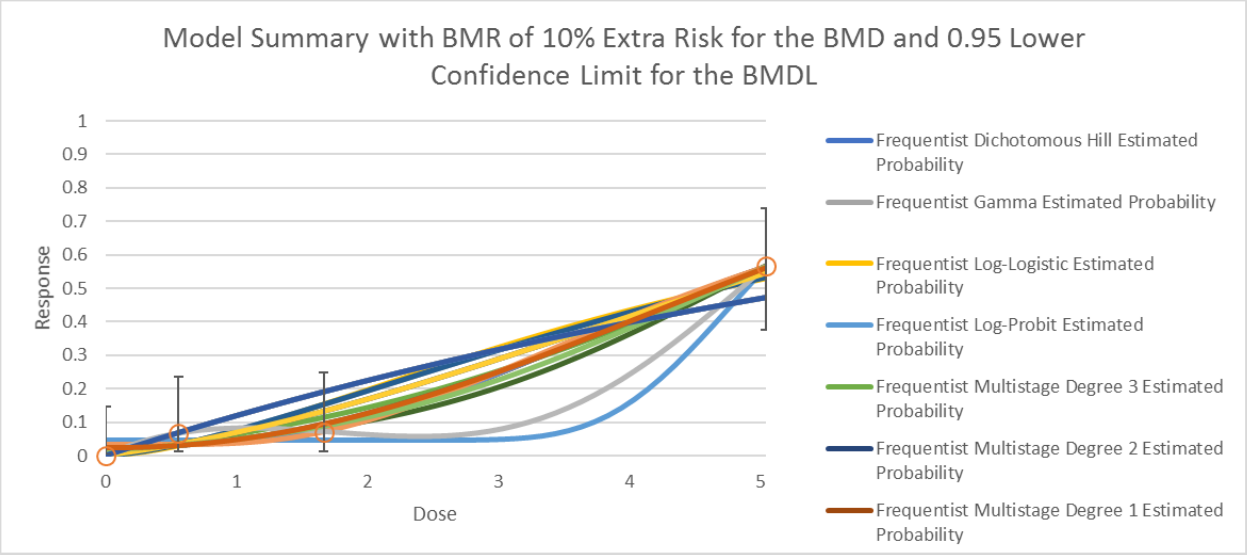

Cavalieri et al. 1983. CPP

Model Name	Posterior Probability	BMD	BMDL	BMDU	P-value	AIC	Scaled residual for dose group nearest the BMD	Scaled residual for control group
Dichotomous Hill	0.04	19.17	12.17	38.70	NA	66.60	-0.03	-1.01
Gamma	0.01	19.13	11.68	29.16	0.15	64.61	-0.01	-1.01
Logistic	0.12	16.56	12.41	21.39	0.32	62.14	-0.51	-0.69
LogLogistic	0.09	19.17	12.17	44.52	0.15	64.60	-0.03	-1.01
LogProbit	0.17	18.60	13.10	41.01	0.15	64.62	0.00	-1.02
Multistage-3	0.01	22.96	2.61	33.83	NA	63.77	0.00	0.00
Multistage-2	0.01	10.69	6.80	21.45	0.12	64.16	-1.53	0.00
Multistage-1	0.01	4.67	3.43	6.54	0.00	74.04	-0.67	0.00
Probit	0.37	14.89	11.27	19.06	0.25	62.42	-0.75	-0.58
Quantal-Linear	0.00	4.67	3.43	6.54	0.00	74.04	-0.67	0.00
Weibull	0.19	19.75	9.43	39.60	0.15	64.56	-0.08	-0.99
**BMA**	-	**15.49**	10.31	22.67	-	-	-	-

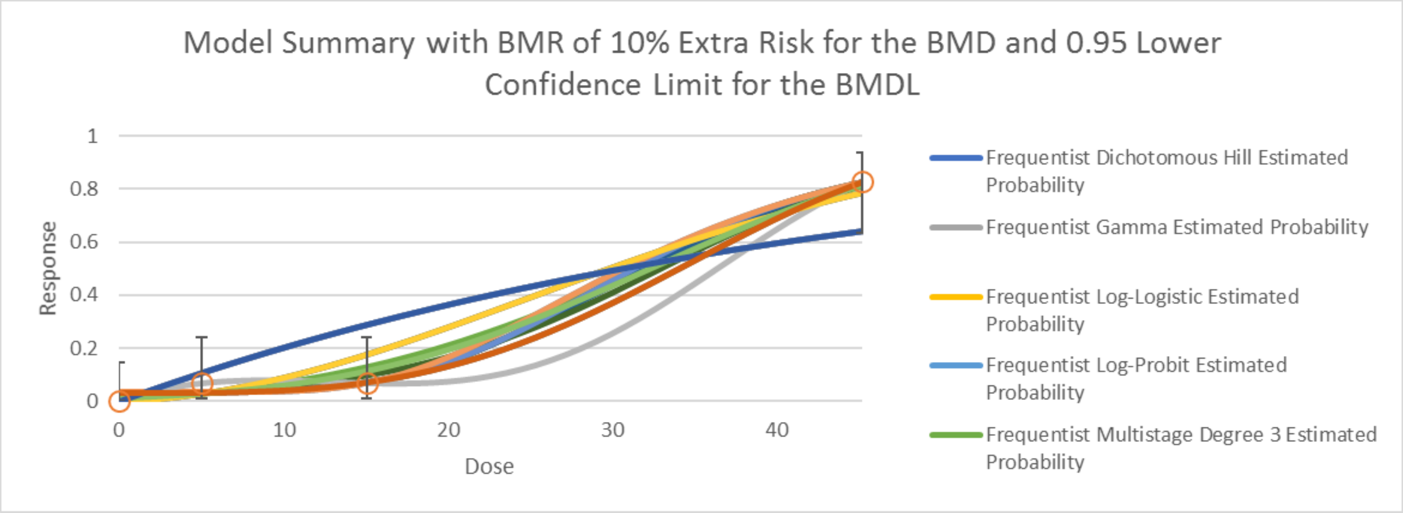

Raveh et al. 1982. BaP

Model Name	Posterior Probability	BMD	BMDL	BMDU	P-value	AIC	Scaled residual for dose group nearest the BMD	Scaled residual for control group
Dichotomous Hill	0.01	2.18	0.52	4.81	0.23	125.26	-0.06	-0.06
Gamma	0.03	0.43	0.00	2.76	0.73	120.88	-0.02	-0.02
Logistic	0.01	4.90	3.83	6.27	0.04	127.22	-1.96	-1.96
LogLogistic	0.00	2.18	0.52	4.81	0.40	123.26	-0.06	-0.06
LogProbit	0.01	2.16	0.51	4.85	0.50	122.35	-0.05	-0.05
Multistage-5	0.00	1.66	1.34	2.50	0.84	119.95	-0.21	-0.21
Multistage-4	0.00	1.72	1.36	2.56	0.74	120.40	-0.25	-0.25
Multistage-3	0.00	1.82	1.39	2.77	0.54	121.33	-0.30	-0.30
Multistage-2	0.00	1.98	1.46	3.21	0.34	122.51	-0.32	-0.32
Multistage-1	0.00	2.16	1.66	2.93	0.43	120.78	-0.43	-0.43
Probit	0.01	5.11	4.12	6.41	0.04	127.82	1.80	-2.05
Quantal-Linear	0.92	2.16	1.66	2.93	0.43	120.78	-0.43	-0.43
Weibull	0.02	0.82	0.10	2.87	0.66	121.20	-0.05	-0.05
**BMA**		**2.38**	1.66	3.45	-	-	-	-

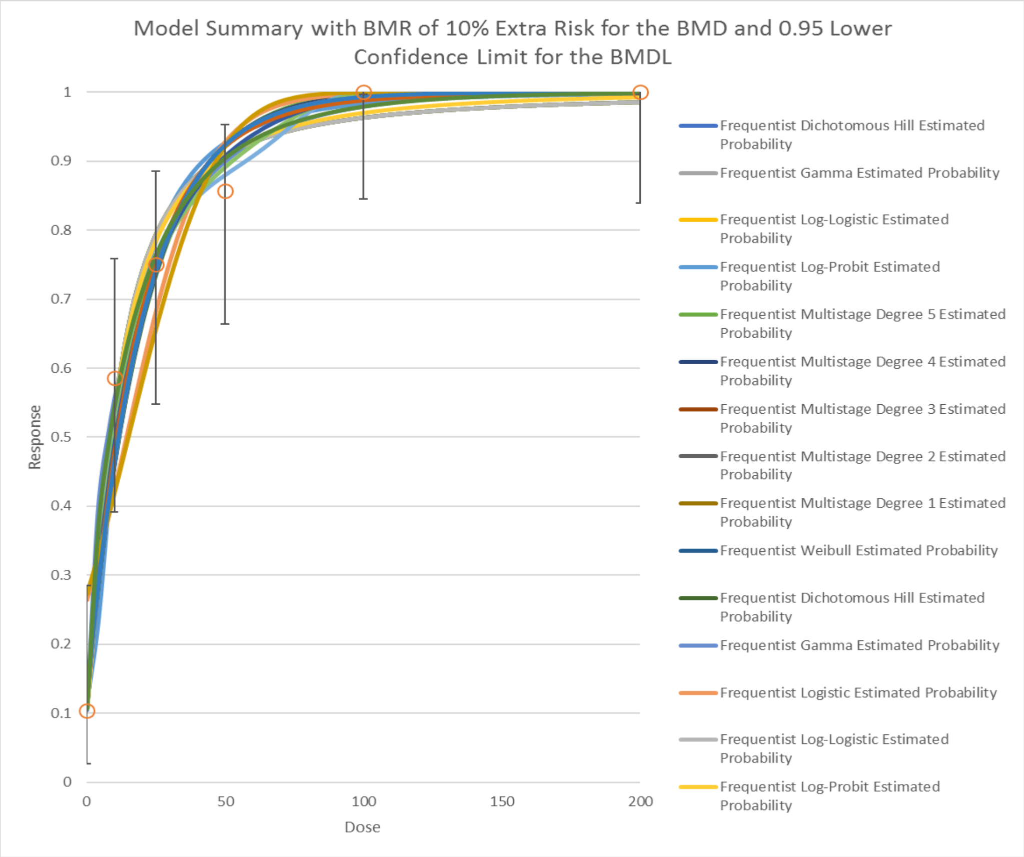

Raveh et al. 1982. CPP

Model Name	Posterior Probability	BMD	BMDL	BMDU	P-value	AIC	Scaled residual for dose group nearest the BMD	Scaled residual for control group
Dichotomous Hill	0.07	33.39	4.84	84.83	NA	122.18	-0.17	0.12
Gamma	0.07	31.74	3.63	85.53	0.71	120.26	-0.23	0.15
Logistic	0.12	53.95	41.99	70.31	0.55	119.29	-0.39	-0.25
LogLogistic	0.13	33.40	4.84	84.83	0.81	120.18	-0.17	0.12
LogProbit	0.04	34.73	5.46	86.07	0.91	120.13	-0.07	0.06
Multistage-3	0.05	46.24	14.27	83.25	NA	122.12	-0.01	0.01
Multistage-2	0.05	27.17	11.27	85.84	0.69	120.28	-0.31	0.22
Multistage-1	0.05	27.62	19.07	44.49	0.92	118.28	-0.30	0.21
Probit	0.13	50.51	39.72	66.36	0.61	119.08	-0.34	-0.18
Quantal-Linear	0.31	27.62	19.07	44.48	0.92	118.28	-0.30	0.21
Weibull	0.08	30.60	4.05	83.95	0.70	120.27	-0.26	0.17
**BMA**	-	**40.72**	19.16	74.16	-	-	-	-

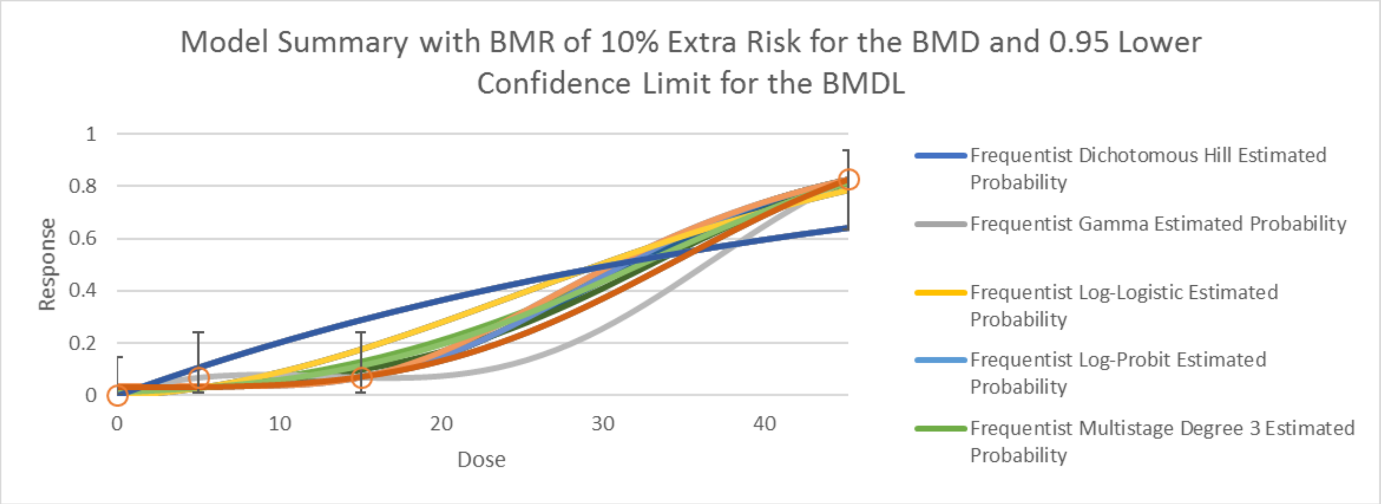

Supplement: Supplementary file 1 [file ijerph-19-09490-s001.zip › ijerph-1775269-supplementary.pdf]
